# Commensal Gut Microbiota Immunomodulatory Actions in Bone Marrow and Liver have Catabolic Effects on Skeletal Homeostasis in Health

**DOI:** 10.1038/s41598-017-06126-x

**Published:** 2017-07-18

**Authors:** Chad M. Novince, Carolyn R. Whittow, Johannes D. Aartun, Jessica D. Hathaway, Nicole Poulides, Michael B. Chavez, Heidi M. Steinkamp, Kaeleigh A. Kirkwood, Emily Huang, Caroline Westwater, Keith L. Kirkwood

**Affiliations:** 1 Department of Oral Health Sciences and Center for Oral Health Research, College of Dental Medicine, Medical University of South Carolina, Charleston, South Carolina 29425 USA; 2 Department of Microbiology and Immunology, Hollings Cancer Center, Medical University of South Carolina, Charleston, South Carolina 29425 USA

## Abstract

Despite knowledge the gut microbiota regulates bone mass, mechanisms governing the normal gut microbiota’s osteoimmunomodulatory effects on skeletal remodeling and homeostasis are unclear in the healthy adult skeleton. Young adult specific-pathogen-free and germ-free mice were used to delineate the commensal microbiota’s immunoregulatory effects on osteoblastogenesis, osteoclastogenesis, marrow T-cell hematopoiesis, and extra-skeletal endocrine organ function. We report the commensal microbiota has anti-anabolic effects suppressing osteoblastogenesis and pro-catabolic effects enhancing osteoclastogenesis, which drive bone loss in health. Suppression of *Sp7*(*Osterix*) and *Igf1* in bone, and serum IGF1, in specific-pathogen-free mice suggest the commensal microbiota’s anti-osteoblastic actions are mediated via local disruption of IGF1-signaling. Differences in the RANKL/OPG Axis *in vivo*, and RANKL-induced maturation of osteoclast-precursors *in vitro*, indicate the commensal microbiota induces sustained changes in RANKL-mediated osteoclastogenesis. Candidate mechanisms mediating commensal microbiota’s pro-osteoclastic actions include altered marrow effector CD4^+^T-cells and a novel Gut-Liver-Bone Axis. The previously unidentified Gut-Liver-Bone Axis intriguingly implies the normal gut microbiota’s osteoimmunomodulatory actions are partly mediated via immunostimulatory effects in the liver. The molecular underpinnings defining commensal gut microbiota immunomodulatory actions on physiologic bone remodeling are highly relevant in advancing the understanding of normal osteoimmunological processes, having implications for the prevention of skeletal deterioration in health and disease.

## Introduction

Gut microbiota interactions with the host modulates gastrointestinal processes, metabolism and immunity^1–5^, having implications for the development and homeostasis of host tissues^6, 7^. Extensive research has focused on the commensal gut microbiota immunoregulatory effects in the context of resistance to pathogenic microbes and intestinal homeostasis^8, 9^, and more recently investigations have begun to define the normal gut microbiota’s role in the pathophysiology of metabolic and autoimmune disease states^4, 6, 9, 10^. Central to this investigation, the commensal gut microbiota’s influence on physiologic tissue remodeling and homeostasis at extra-gastrointestinal sites is largely unknown^11^.

The study of osteoimmunology has elucidated that innate-immunity, marrow effector T-cells, and diverse endocrine organs regulate osteoclast-osteoblast mediated bone remodeling, both in health and disease^12–17^. Bone remodeling is a continuous dynamic skeletal renewal process in which monocyte-myeloid derived osteoclast cells resorb old bone matrix, and mesenchymal derived osteoblast cells subsequently form new bone matrix. Skeletal homeostasis occurs when balanced osteoclast-osteoblast actions remodel the adult skeleton, and there is no net gain/loss of osseous tissue^12–14, 18^. Understanding that resident gut microbes significantly modulates host immunity^1–5^ and metabolic-endocrine processes^4, 6, 7^ in health, the commensal gut microbiota is a strong candidate immunoregulator of physiologic bone remodeling processes in the adult skeleton.

The impact of resident gut microbes on skeletal physiology was introduced by two early life antibiotic treatment studies^19, 20^, and a growing Conventional (Conv) vs. germ-free (GF) mouse model investigation^21^, which revealed the normal gut microbiota has inhibitory effects on the accrual of bone mass in the post-natal developing skeleton of C57BL/6 mice. Subsequent GF mouse model investigations demonstrating that the normal gut microbiota supports skeletal growth and bone formation in BALB/c^22^ and CB6F1^23^ mice, notably imply that the commensal microbiota impact on skeletal physiology is regulated by mouse strain genetic determinants. While resident gut microbes have been reported to have pro-osteoclastic immunomodulatory effects impairing the accrual of bone mass in growing C57BL/6 mice^21^, and pro-anabolic actions enhancing liver IGF1 mediated skeletal growth in BALB/c^22^ and CB6F1^23^ mice, the normal gut microbiota’s osteoimmunomodulatory effects on skeletal physiology are largely unknown in the healthy adult skeleton.

This osteoimmunology study in young adult C57BL/6 SPF vs. GF mice introduces the commensal gut microbiota as a potent immunoregulator of osteoclast-osteoblast mediated bone remodeling processes in the healthy adult skeleton. 3 month-old mice were investigated since this is the age when bone modeling (growth) is considered principally complete, and physiologic bone remodeling (turnover) is highly metabolically active in the C57BL/6 strain^24, 25^. Appreciating that the adult skeleton continues to accrue peak bone mineral content until about age 30 years in humans^26, 27^, optimizing skeletal remodeling processes during young adulthood has significant implications for the prevention of osteoporosis and other aging associated skeletal deterioration states.

While experimental disease studies have elucidated that the commensal gut microbiota drives skeletal deterioration in pathophysiologic states^28–30^, this report reveals the commensal gut microbiota has pro-osteoclastic and anti-osteoblastic actions which drive catabolic effects on skeletal tissue homeostasis in health. Identification of a novel Gut-Liver-Bone Axis intriguingly implies the commensal gut microbiota’s catabolic actions on physiologic skeletal homeostasis are in part mediated by pro-inflammatory immunostimulatory effects in liver. Having broad systemic implications, this investigation highlights the commensal gut microbiota as a critical regulator of physiologic tissue remodeling and homeostasis at extra-gastrointestinal sites^11^, a biological phenomenon which is currently unappreciated and not well understood.

## Results

### Commensal microbiota has anti-anabolic effects on trabecular bone remodeling

**Figure 1:**
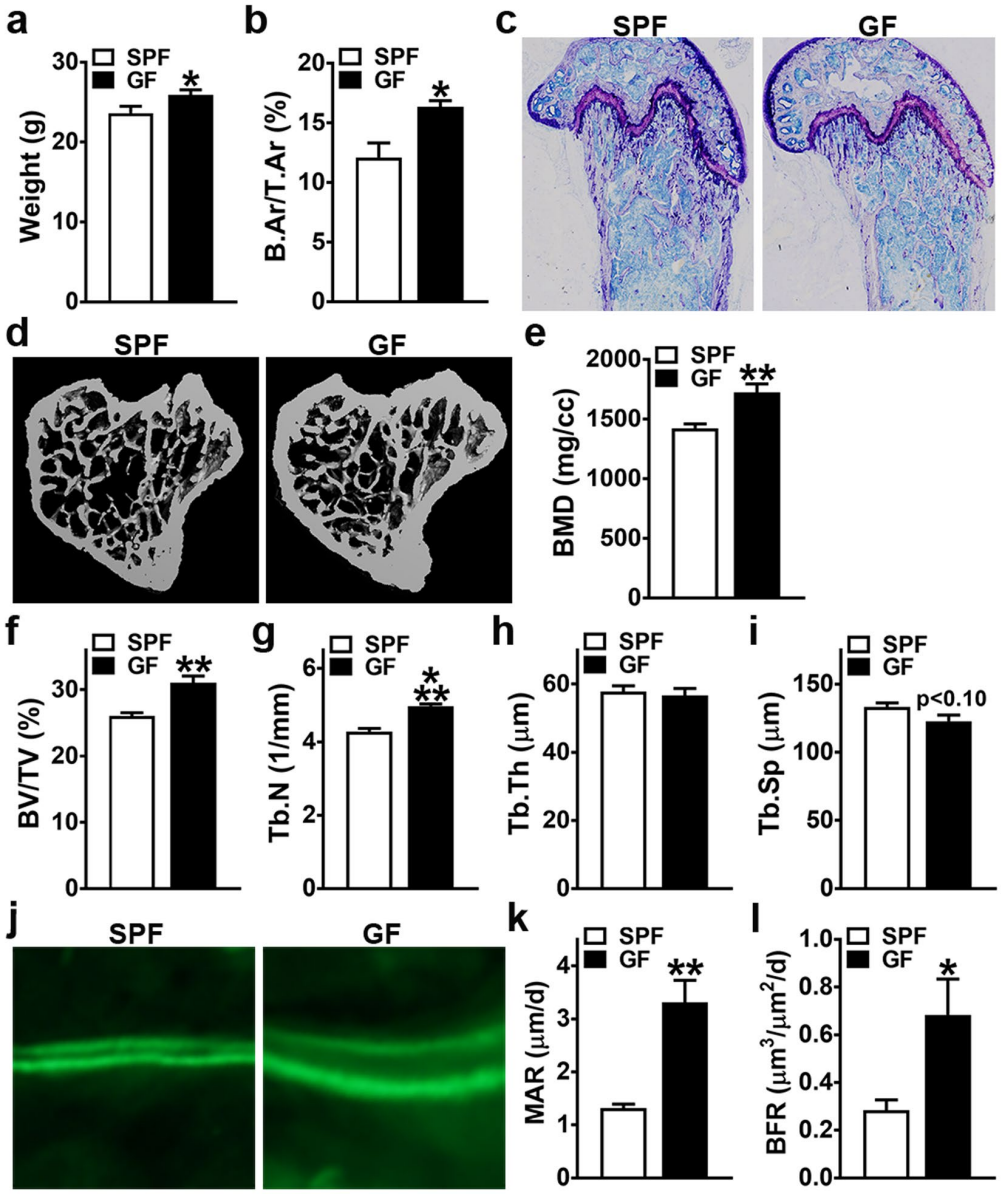
Animal weight and trabecular bone analysis. 11 to 12 week-old male SPF & GF mice were weighed; euthanized; femur harvested for histomorphometric analysis and tibia harvested for micro-CT analysis. (**a**) Animal weight (n = 12/gp). (**b**,**c**) Static histomorphometric analysis of distal femur trabecular bone area (n = 4/gp). (**b**) B.Ar/T.Ar = bone area. (**c**) Representative images (100×) of toluidine blue stained distal femur sections. (**d**–**i**) Micro-CT analysis of proximal tibia trabecular bone (n = 4/gp). (**d**) Representative reconstructed cross-sectional images, extending 360 µm distally from where analysis was initiated. (**e**) BMD = trabecular bone mineral density. (**f**) BV/TV = trabecular bone volume fraction. (**g**) Tb.N = trabecular number. (**h**) Tb.Th = trabecular thickness. (**i**) Tb.Sp = trabecular separation. (**j**–**l**) Dynamic histomorphometric analysis of bone formation indices in distal femur trabecular bone; calcein administered 5 and 2 days prior to sacrifice (n = 4/gp). (**j**) Representative images of calcein labeled secondary spongiosa (400×). (**k**) MAR = mineral apposition rate. (**l**) BFR = bone formation rate. Data reported as mean ± SEM. *p < 0.05 vs. SPF; **p < 0.01 vs. SPF; ***p < 0.001 vs SPF.

Histomorphometric and micro-CT studies were carried out in the trabecular bone compartment, based on the anatomical region being the most metabolically active site of bone remodeling in the young adult bone organ^31, 32^. Histomorphometric analysis revealed decreased trabecular bone area (B.Ar/T.Ar) in the distal femur of SPF vs. GF mice (Fig. 1b,c). Micro-CT analysis of the proximal tibia demonstrated an osteopenic trabecular bone phenotype in SPF vs. GF mice (Fig. 1e). Consistent with the reduced trabecular bone quantity found in the distal femur via histomorphometry (Fig. 1b), micro-CT analysis revealed decreased trabecular bone volume (BV/TV) in the proximal tibia of SPF mice (Fig. 1f). Inferior trabecular bone micro-architecture properties in the proximal tibia of SPF mice were characterized by reduced trabecular number (Tb.N) (Fig. 1g), and a trend towards increased trabecular separation (Tb.Sp) (Fig. 1i). Micro-CT analysis of cortical bone parameters revealed no differences in femur length, and marginally reduced cortical bone (Ct.Ar/Tt.Ar) in the femur mid-diaphysis of SPF mice (Supplementary Fig. S1).

Recognizing that the normal gut microbiota blunts the accrual of bone mass during bone modeling (growth) processes in the developing C57BL/6 skeleton^19–21^, dynamic bone formation indices were analyzed (Fig. 1j–l) to validate that alterations in bone remodeling are contributing to the osteopenic trabecular skeletal phenotype found in young adult SPF vs. GF mice. Mineral apposition rate (MAR) (Fig. 1k) and bone formation rate (BFR) (Fig. 1l) were suppressed in the distal femur trabecular bone of SPF mice, which demonstrates that the commensal gut microbiota has anti-anabolic effects on trabecular bone remodeling in the adult skeleton.

### Commensal microbiota suppresses osteoblast differentiation and function

**Figure 2:**
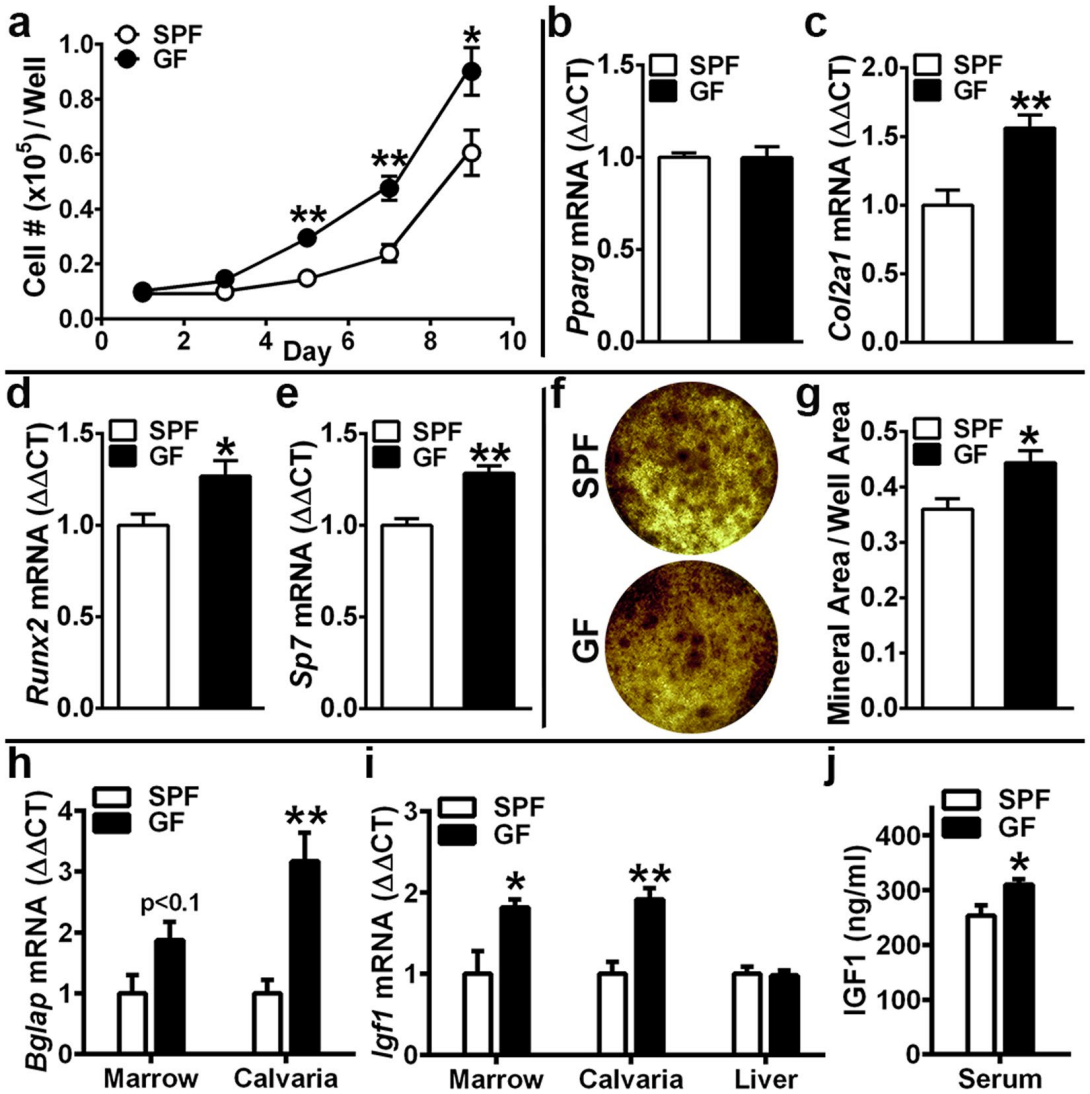
Osteoblastogenesis investigations. (**a**–**g**) Bone marrow stromal cell (BMSC) *in vitro* osteoblastogenesis assays. 11 week-old male SPF & GF mice were euthanized; bone marrow harvested; BMSCs isolated for *in vitro* assays. (**a**) Cell expansion assay (n = 4/gp): cell numbers over time in culture. (**b**–**e**) BMSC differentiation potential assay (untreated day-4 pre-confluent cultures were harvested for qRT-PCR analysis) to assess intrinsic differentiation potential (n = 4/gp). (**b**) *Pparg* mRNA assessed as a marker of adipogenic potential. (**c**) *Col2a1* mRNA assessed as a marker of chondrogenic potential. (**d**) *Runx2* and (**e**) *Sp7(Osterix)* mRNA assessed as markers of osteoblastogenic potential. Relative quantification of mRNA was performed via the comparative *C*_T_ method (ΔΔCT); *Gapdh* was utilized as an internal control gene; data expressed as fold difference relative to SPF. (**f**,**g**) von Kossa mineralization assay (21 day mineralization treatment) (n = 4/gp). (**f**) Representative von Kossa stained culture images. (**g**) Mineralization area per well area. (**a**–**g**) BMSC assays carried out in duplicate (two technical replicate) cultures; n-values represent biological replicates per group. (**h**–**j**) Commensal microbiota *in vivo* regulation of osteoblastogenesis. 11 to 12 week-old male SPF & GF mice were euthanized; tissues were harvested. (h,i) RNA was isolated from marrow (n = 4/gp), calvaria (n = 4/gp), liver (n = 6/gp) for qRT-PCR analysis of candidate osteogenic genes. (**h**) *Bglap(Osteocalcin)* mRNA assessed as a marker of mature osteoblast function, and (**i**) *Igf1* mRNA assessed as a critical osteoblastic signaling factor. Relative quantification of mRNA was performed via the comparative *C*_T_ method (ΔΔCT); *Gapdh* was utilized as an internal control gene; data expressed as fold difference relative to SPF. (**j**) Serum was isolated from whole blood (n = 10/gp); ELISA analysis of IGF1 levels. Data reported as mean ± SEM. *p < 0.05 vs. SPF; **p < 0.01 vs. SPF.

Considering the blunted dynamic bone formation indices found in the remodeling trabecular bone of SPF mice (Fig. 1j–l), bone marrow stromal cell (BMSC) osteoblast-progenitors were isolated to elucidate commensal microbiota induced alterations in osteoblast potential (Fig. 2a–g). BMSC expansion over time was blunted in SPF vs. GF BMSC cultures (Fig. 2a), and gene expression studies in untreated day-4 BMSC cultures revealed alterations in intrinsic mesenchymal-stromal cell differentiation potential (Fig. 2b–e). *Col2a1* (Fig. 2c), an early marker for chondrogenesis, and *Runx2* (Fig. 2d) and *Sp7* (Fig. 2e), transcription factors critical for commitment to and maturation in the osteoblastic lineage^33^, were significantly decreased in day-4 BMSC cultures from SPF vs. GF mice. Consistent with the reduced osteoblastic differentiation potential observed in untreated day-4 BMSC cultures from SPF mice (Fig. 2d,e), 21-days mineralization treatment (von Kossa assay) induced less mineralization in SPF vs. GF BMSC cultures (Fig. 2f,g).

### SPF mice have decreased *Bglap* and *Igf1* in bone, and lower serum IGF1

In light of the blunted mineralization in SPF trabecular bone (Fig. 1k) and BMSC cultures (Fig. 2f,g), *Bglap*(*Osteocalcin*) was assessed as a marker for mature osteoblast function in bone marrow and calvaria (Fig. 2h). While bone marrow and calvaria are both heterogeneous in their cellular composition, the rationale for including calvaria is based on its more homogeneous stromal-osteoblastic cellular composition better reflecting osteoblast specific gene expression. Corroborating the suppressed osteoblast function detected *in vivo* (Fig. 1k) and *in vitro* (Fig. 2f,g) in SPF vs. GF mice, *Bglap* was marginally down-regulated in SPF bone marrow, and significantly decreased in SPF calvaria (Fig. 2h).

Considering *Sp7*(*Osterix*) was decreased in BMSC cultures from SPF mice (Fig. 2e), and that IGF1 signaling upregulates *Sp7* mediated osteoblast maturation and function^33, 34^, *Igf1* expression and serum IGF1 were evaluated (Fig. 2i,j). *Igf1* was decreased in SPF vs. GF bone marrow and calvaria (Fig. 2i), which suggest the commensal gut microbiota’s inhibitory effects on osteoblastogenesis are potentially mediated through blunted IGF1 signaling in osteoblastic cells^35^. Indirect evidence from a transgenic mouse model locally deficient in IGF1 signaling within osteoblastic cells, which has a skeletal phenotype (reduced BV/TV, decreased Tb.N, increased Tb.Sp)^35^ strikingly similar to SPF vs. GF mice (Fig. 1), indicates the commensal gut microbiota impact on trabecular bone morphology may occur in part by impaired IGF1 signaling within skeletal tissue. Consistent with the reduced *Igf1* expression in SPF vs. GF bone tissues (Fig. 2i), serum IGF1 was 18.3% lower in SPF mice (Fig. 2j). Appreciating that liver-derived IGF1 constitutes 70% of circulating IGF1^36^, differences were ruled out in liver *Igf1* expression (Fig. 2i). While the suppressed osteoblastogenesis phenotype in SPF vs GF mice (Fig. 1j–l; Fig. 2) delineates the commensal microbiota’s catabolic effects on skeletal remodeling are mediated in part by blunted osteoblast bone formation, recognizing that bone remodeling occurs through dual osteoclast-osteoblast actions, investigations were carried out to elucidate the commensal microbiota impact on osteoclastogenesis.

### Commensal microbiota enhances osteoclast size and eroded bone perimeter

**Figure 3:**
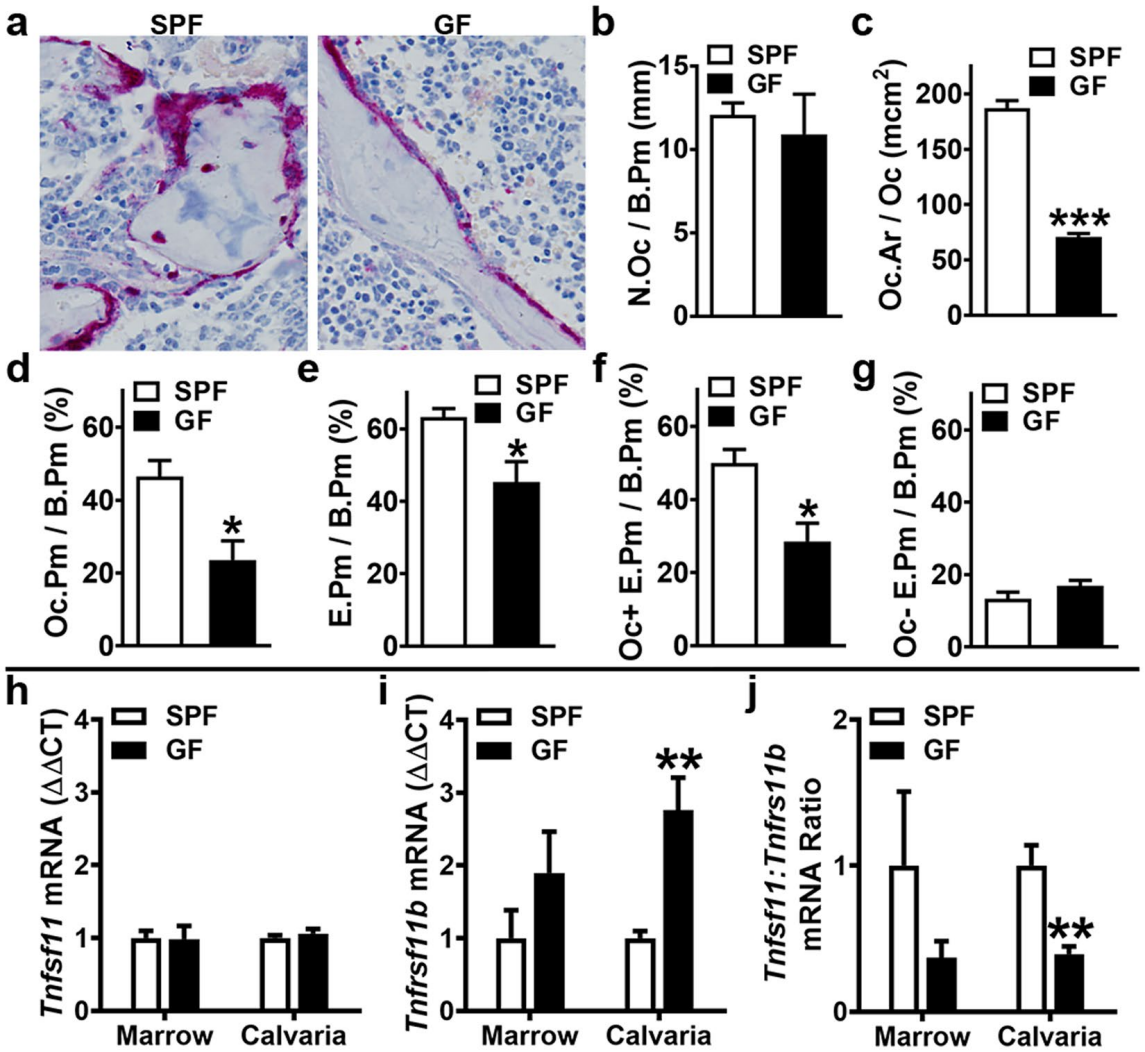
Commensal microbiota *in vivo* regulation of osteoclastogenesis. 12 week-old male SPF & GF mice were euthanized; (**a**–**g**) femurs harvested for histomorphometric analyses (n = 4/gp), and (**h**–**j**) bone marrow and calvaria were harvested for gene expression analysis (n = 4/gp). (**a**–**g**) Histomorphometric analyses of osteoclast cellular endpoints and resorbed bone were performed in the trabecular bone secondary spongiosa of tartrate-resistant acid phosphatase (TRAP) stained distal femur sections; TRAP + cell lining bone with ≥ 3 nuclei designated an osteoclast. (**a**) Representative images of TRAP-stained secondary spongiosa (400×). (**b**) N.Oc/B.Pm = osteoclast number per bone perimeter. (**c**) Oc.Ar/Oc = average osteoclast area. (**d**) Oc.Pm/B.Pm = osteoclast perimeter per bone perimeter. (**e**) E.Pm/B.Pm = eroded perimeter per bone perimeter. (**f**) Oc + E.Pm/B.Pm = osteoclast-positive eroded perimeter per bone perimeter. (**g**) Oc-E.Pm/B.Pm = osteoclast-negative eroded perimeter per bone perimeter. (**h**–**j**) qRT-PCR analysis in bone marrow and calvaria to assess alterations in the RANKL/OPG Axis. (**h**) *Tnfsf11(Rankl)* mRNA in bone marrow and calvaria. (**i**) *Tnfrsf11b(Opg)* mRNA in bone marrow and calvaria. (**j**) *Tnfsf11(Rankl)*:*Tnfrsf11b(Opg)* ratio in bone marrow and calvaria. Relative quantification of mRNA was performed via the comparative *C*_T_ method (ΔΔCT); *Gapdh* was utilized as an internal control gene; data expressed as fold difference relative to SPF. Data reported as mean ± SEM. *p < 0.05 vs. SPF; **p < 0.01 vs SPF; ***p < 0.001 vs SPF.

Histomorphometric analysis of tartrate-resistant acid phosphatase (TRAP) stained distal femur sections was performed to investigate the commensal microbiota’s effects on *in vivo* osteoclastogenesis (Fig. 3a–g). SPF vs. GF mice had similar numbers of osteoclasts lining the trabecular bone perimeter (N.Oc/B.Pm) (Fig. 3b), which suggest the commensal microbiota does not alter the commitment of monocyte-myeloid cells to the osteoclast lineage. The average osteoclast cell size (Oc.Ar/Oc) was 2.5× larger in SPF mice (Fig. 3c), which resulted in a 2× greater osteoclast perimeter per bone perimeter (Oc.Pm/B.Pm) in SPF vs. GF mice (Fig. 3d). The substantially increased Oc.Ar/Oc (Fig. 3c) in SPF mice, implies the commensal microbiota enhances osteoclast maturation.

Eroded bone perimeter analysis (Fig. 3e–g) was performed to assess alterations in osteoclast function. SPF vs. GF mice had an increased eroded perimeter per bone perimeter (E.Pm/B.Pm) (Fig. 3e), demonstrating that the commensal microbiota upregulates bone resorption in health. Findings that the increased osteoclast-positive eroded perimeter per bone perimeter (Oc + E.Pm/B.Pm) (Fig. 3f), paralleled the 2× greater Oc.Pm/B.Pm (Fig. 3d) in SPF vs. GF mice, indicates the commensal microbiota’s pro-resorptive actions are attributed to mechanisms enhancing osteoclast size/maturation (Fig. 3c).

### Commensal microbiota modulates the RANKL/OPG Axis

Based on the *in vivo* histomorphometry findings revealing enhanced osteoclast size/maturation in SPF mice (Fig. 3c), the *Tnfsf11(Rankl)/Tnfrsf11b(Opg)* Axis was assessed (Fig. 3h–j) to evaluate alterations in critical osteoclastic signaling factors^13, 15, 18^. RANKL, which signals at the RANK receptor on pre-osteoclast/osteoclast cells, is required for osteoclast differentiation/maturation. Due to OPG functioning as the RANK decoy receptor, it is imperative to assess the RANKL/OPG ratio when evaluating RANKL levels. Gene expression analysis revealed a trend towards a higher *Tnfsf11*:*Tnfrsf11b* ratio in marrow, and a significantly upregulated *Tnfsf11*:*Tnfrsf11b* ratio in calvaria of SPF vs. GF mice (Fig. 3j). The increased *Tnfsf11*:*Tnfrsf11b* ratio findings in SPF mice (Fig. 3j) were interestingly attributed to enhanced *Tnfrsf11b* expression (Fig. 3i), not alterations in *Tnfsf11* expression (Fig. 3h). Recognizing that *Tnfrsf11b* is primarily expressed by stromal-osteoblastic cells in the bone environment, the findings that *Tnfrsf11b* upregulation is a trend in bone marrow and significant in calvaria (Fig. 3i) are in-line with calvaria vs. marrow having a more homogeneous stromal-osteoblastic cellular composition. Appreciating that an increased *Tnfsf11*:*Tnfrsf11b* ratio indicates higher levels of unbound RANKL available in the bone environment to activate RANK signaling, this notably implies that differences in *in vivo* RANKL signaling contribute to the superior osteoclast size/maturation phenotype in SPF vs. GF mice (Fig. 3c).

### Commensal microbiota dynamically regulates osteoclast-precursor cell maturation

**Figure 4:**
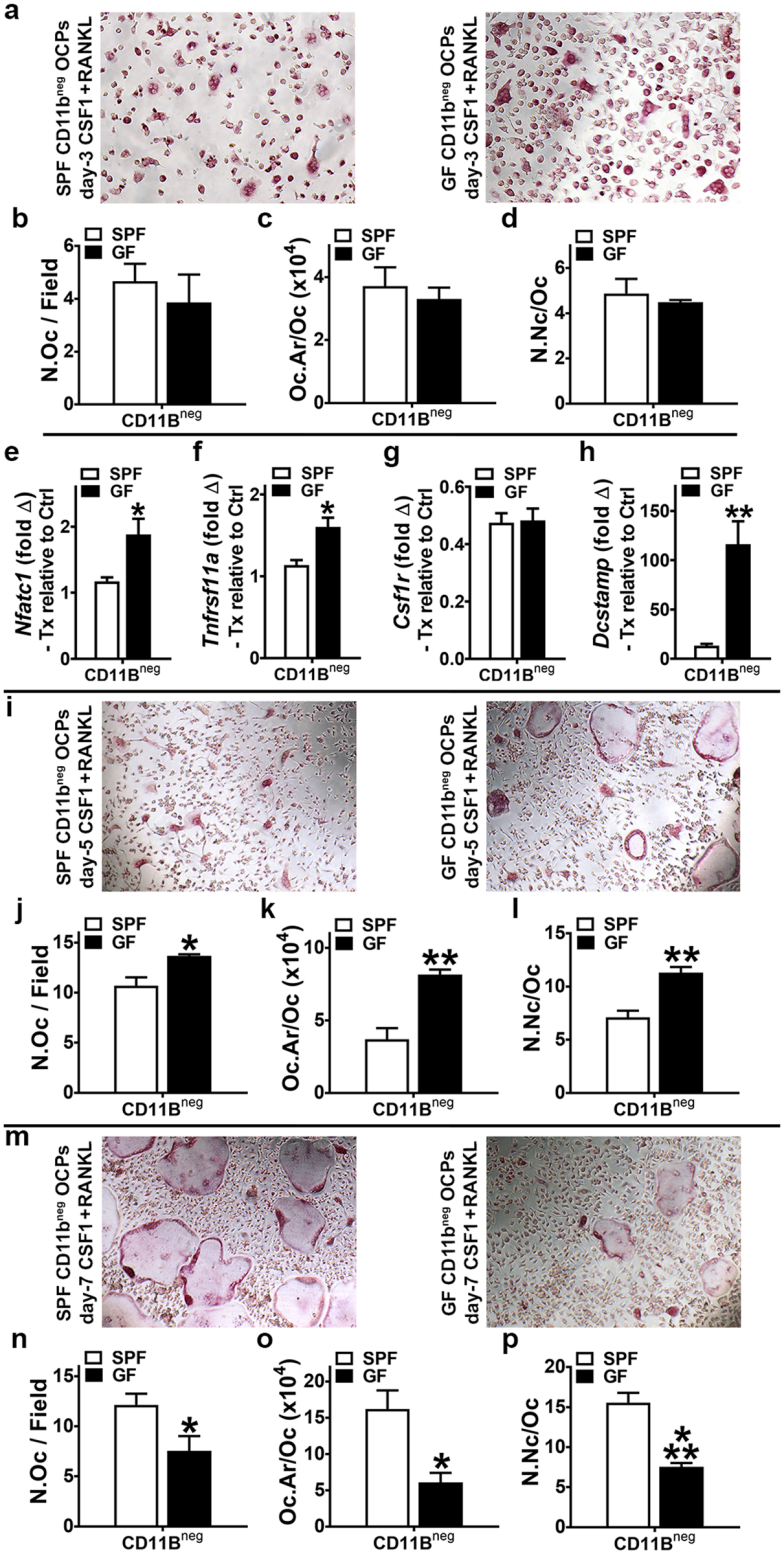
Osteoclast-precursor (OCP) differentiation assays. (**a**–**p**) 11 week-old male SPF & GF mice were euthanized; bone marrow harvested; hematopoietic progenitor cells (HPCs) isolated. Magnetic cell sorting was applied to separate CD11b^neg^ HPCs, which were then stimulated in culture (primed with CSF1) to enrich for CD11b^neg^ osteoclast-precursor (OCP) cells having high osteoclastic potential. CD11b^neg^ OCP cultures were then stimulated with control (CSF1 alone) or treatment (CSF1 & RANKL) media for 3, 5 and 7 days. Cytomorphometric cellular differentiation endpoints were analyzed in TRAP stained CD11b^neg^ OCP cultures at day-3, day-5, and day-7 to evaluate cell level alterations in RANKL-induced osteoclast differentiation; TRAP + cell with > 3 nuclei considered an osteoclast. Gene expression studies were carried out in CD11b^neg^ OCP cultures at day-3 to detect early transcription level alterations in RANKL-stimulated osteoclast differentiation. (**a**–**d**) Day-3 TRAP stain assay (n = 4/gp). (**a**) Representative images (200X) of CD11b^neg^ OCP cultures stimulated with treatment (CSF1 & RANKL) media for 3 days. (**b**) N.Oc/Field = number of osteoclasts per field of view. (**c**) Oc.Ar/Oc = average osteoclast area. (**d**) N.Nc/Oc = nuclei number per osteoclast. (**e**–**h**) Day-3 qRT-PCR gene expression assay (n = 4/gp). (**e**) *Nfatc1* mRNA. (**f**) *Tnfrsf11a* mRNA. (**g**) *Csf1r* mRNA. (**h**) *Dcstamp* mRNA. Relative quantification of mRNA was performed via the comparative *C*_T_ method (ΔΔCT); *Gapdh* was utilized as an internal control gene; data expressed as treatment (CSF1 and RANKL) fold change relative to control (CSF1). (**i**–**l**) Day-5 TRAP stain assay (n = 4/gp). (**i**) Representative images (100×) of CD11b^neg^ OCP cultures stimulated with treatment (CSF1 & RANKL) media for 5 days. (**j**) N.Oc/Field. (**k**) Oc.Ar/Oc. (**l**) N.Nc/Oc. (**m**–**p**) Day-7 TRAP stain assay (n = 4/gp). (**m**) Representative images (100×) of CD11b^neg^ OCP cultures stimulated with treatment (CSF1 & RANKL) media for 7 days. (**n**) N.Oc/Field. (**o**) Oc.Ar/Oc. (**p**) N.Nc/Oc. Gene expression assay performed in duplicate (two technical replicate) cultures. TRAP stain assays performed in triplicate (three technical replicate) cultures; three fields of view analyzed per technical replicate culture. n-values represent biological replicates per group. Data reported as mean ± SEM. *p < 0.05 vs. SPF; **p < 0.01 vs. SPF; ***p < 0.001 vs SPF.

Osteoclast-precursor (OCP) *in vitro* differentiation assays (Fig. 4a–p) were utilized to further define the commensal microbiota’s regulatory effects on osteoclast maturation, including the role of RANKL-signaling. Isolated marrow hematopoietic progenitor cells (HPCs) were labeled with CD11b MicroBeads, magnetic cell sorting was applied to separate CD11b^neg^ HPCs, and cells were stimulated in culture (primed with CSF1) to enrich for CD11b^neg^ osteoclast-precursor (OCP) cells having high osteoclastic potential^37–39^. CD11b^neg^ OCP cultures were then subjected to stimulation with control (CSF1 alone) or treatment (CSF1 & RANKL) media for 3, 5 and 7 days^37–39^. Cultures stimulated for 3, 5 and 7 days were TRAP stained for cytomorphometric analysis of cellular differentiation endpoints (Fig. 4; Supplementary Fig. S2), to evaluate cell level alterations in RANKL-induced osteoclast differentiation. Appreciating that day-3 of the culture system is when OCPs begin fusing to form multi-nucleated osteoclasts^37–39^, gene expression studies were carried out in day-3 OCP cultures (Fig. 4e–h) to detect early transcription level alterations in RANKL-stimulated osteoclast differentiation.

Cytomorphometric analysis of day-3 OCP cultures showed no differences in SPF vs. GF OCP cellular endpoints (Fig. 4a–d), which implies commensal microbiota immunomodulatory effects do not alter RANKL-induced early commitment of pre-osteoclastic cells to the osteoclast lineage. Day-3 OCP culture gene expression analysis revealed alterations in RANKL-stimulated osteoclastic genes, which unexpectedly indicated enhanced cell level differentiation potential outcomes in GF vs. SPF OCPs at later time points in the culture system. *Nfatc1* (Fig. 4e), the master transcription factor for osteoclastogenesis, and *Tnfrsf11a* (Fig. 4f), the RANKL receptor and a surrogate marker for *in vitro* osteoclast maturation, were decreased in SPF vs. GF CD11b^neg^ OCPs. Appreciating that signaling at both the CSF1R and RANK receptor are critical and necessary for osteoclast differentiation, differences in *Csf1r* (Fig. 4g) were ruled out to delineate that alterations in SPF vs. GF OCP outcomes are mediated by RANKL-stimulation.

Day-5 OCP culture cytomorphometric analysis elucidated cell level alterations in OCP differentiation potential (Fig. 4i–l), which notably paralleled the *Nfatc1* (Fig. 4e) and *Tnfrsf11a* (Fig. 4f) gene expression level differences found in day-3 cultures. Day-5 OCP culture osteoclast size (Oc.Ar/Oc) (Fig. 4k) and numbers of nuclei per osteoclast (N.Nc/Oc) (Fig. 4l) were decreased in SPF vs. GF CD11b^neg^ OCP cultures. Cytomorphometric analysis of day-7 OCP cultures (Fig. 4m–p), which reflects terminal osteoclast differentiation potential in the OCP culture system, validated the observed superior *in vivo* osteoclast maturation phenotype noted in distal femur trabecular bone of SPF mice (Fig. 3c). The 2.5× larger osteoclast size (Oc.Ar/Oc) lining the trabecular bone in SPF mice (Fig. 3c), was remarkably paralleled by 2.5× larger Oc.Ar/Oc in SPF vs. GF OCP cultures (Fig. 4o). Number of nuclei per osteoclast (N.Nc/Oc), another indicator of osteoclast maturation, was also greater in day-7 SPF OCP cultures (Fig. 4p).

The study of an enriched OCP population (CD11b^neg^) facilitated elucidating subtle alterations in the osteoclastogenic capacity of SPF vs. GF mice, which appears to be secondary to commensal microbiota immunoregulatory effects modulating RANKL-induced early osteoclast fusion genes. *Dcstamp*, a transmembrane protein critical for osteoclast fusion which is upregulated by RANKL-signaling^40^, was profoundly increased in day-3 GF OCP cultures (Fig. 4h). Appreciating that *Dcstamp* is essential for RANKL-induced osteoclastogenesis^40^, and considering *Dcstamp* was substantially increased in CD11b^neg^ OCPs from GF vs. SPF mice (Fig. 4h), it appears the absence of the commensal microbiota *in vivo* immunostimulation sensitizes CD11b^neg^ OCPs to RANKL-stimulated osteoclast differentiation/maturation. The realization that the enhanced osteoclast differentiation endpoints found in the GF vs. SPF OCP cultures at day-5 (Fig. 4k,l) were lost at day-7 (Fig. 4o,p), suggests that the sensitized RANKL-signaling in GF CD11b^neg^ OCPs was transient.

The altered susceptibility of SPF vs. GF OCPs to RANKL stimulation in the OCP culture system provides novel insight into the commensal microbiota immunomodulatory impact on physiologic osteoclastogenesis. Osteoclasts are critical for normal skeletal turnover and repair processes, which is evidenced by anti-resorptive medications’ deleterious effects (osteonecrosis of the jaw, atypical femoral fractures). Considering that RANKL is locally upregulated at osseous micro-damage^41^ and early fracture sites^42^ to transiently stimulate osteoclastic bone resorption necessary for normal osseous repair processes, based on the finding that RANKL-stimulation induced a more rapid onset and shorter duration pro-osteoclastogenic effects in GF vs. SPF OCPs (Fig. 4), the absence of the commensal microbiota may be advantageous for acute skeletal repair. Recognizing that increased chronic systemic inflammation has catabolic effects on systemic skeletal remodeling^12^ and disrupts local osseous tissue repair^43^, the enhanced terminal osteoclast maturation phenotype shown in SPF mice (Fig. 3c; Fig. 4m–p) implies that commensal microbiota pro-osteoclastic immunostimulatory effects are detrimental to skeletal tissue homeostasis in health.

### SPF mice have increased pro-inflammatory cytokines in marrow & liver, and higher serum TNF

**Figure 5:**
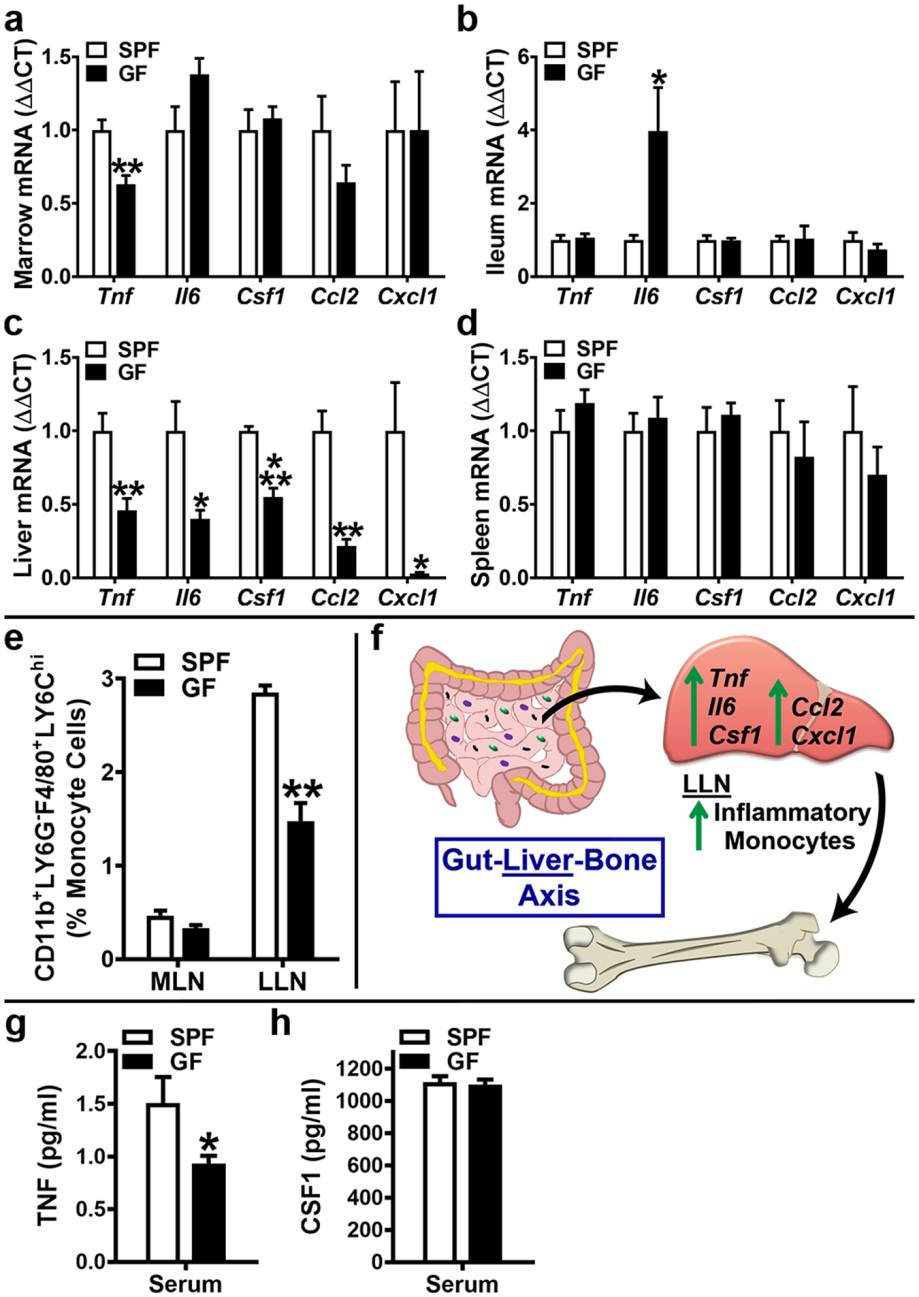
Commensal microbiota *in vivo* regulation of pro-inflammatory cytokines. 11 to 12 week-old male SPF & GF mice were euthanized; tissues harvested for (**a**–**d**) gene expression assays, (**e**) flow cytometry assays, and (**g**,**h**) ELISA assays. (**a**–**d**) RNA was isolated from tissues and qRT-PCR analysis was performed in (**a**) bone marrow (n = 4/gp), (**b**) ileum (n = 6/gp), (**c**) liver (n = 6/gp), (**d**) spleen (n = 4/gp) to assess *Tnf*, *Il6*, *Csf1*, *Ccl2*, *Cxcl1* mRNA. Relative quantification of mRNA was performed via the comparative *C*_T_ method (ΔΔCT); *Gapdh* was utilized as an internal control gene; data expressed as fold difference relative to SPF. (**e**) Mesenteric lymph node (MLN) cells and liver lymph node (LLN) cells were isolated and stained for flow cytometric analysis (n = 4/gp) to assess the frequency of CD11b^+^LY6G^-^F4/80^+^LY6C^hi^ (inflammatory monocyte) cells. Cell percentages are expressed relative to total gated monocyte cells. (**f**) Schematic of newly identified/proposed Gut-Liver-Bone Axis. (**g**,**h**) Serum was isolated from whole blood (n = 9–10/gp); ELISA analysis of (**g**) TNF levels and (**h**) CSF1 levels. Data are reported as mean ± SEM. *p < 0.05 vs. SPF; **p < 0.01 vs. SPF; ***p < 0.001 vs. SPF.

In order to further elucidate osteoimmunoregulatory mechanisms mediating the commensal gut microbiota’s actions supporting terminal osteoclast maturation (Fig. 3c; Fig. 4o,p), the expression of candidate pro-inflammatory innate-immune cytokines were assessed in bone marrow (Fig. 5a) and at hepato-gastrointestinal sites (Fig. 5b–d). *Tnf*, a potent pro-inflammatory cytokine which enhances RANKL-mediated osteoclastogenesis and inhibits osteoblastogenesis^18, 44^, was increased in SPF vs. GF marrow (Fig. 5a).

Due to the proximity of the resident gut microbiota, upregulated cytokine expression was anticipated in the ileum of SPF mice. There was no difference in *Tnf*, *Csf1*, *Ccl2* or *Cxcl1* and surprisingly *Il6* was elevated in the GF ileum (Fig. 5b). The increased *Il6* expression in the ileum of GF vs. SPF mice (Fig. 5b) is consistent with a prior report in the colon of GF vs. Conv mice^21^, which implies that in absence of exogenous gut microbiota immuno-stimulation, *Il6* is endogenously upregulated to support gut homeostasis/function.

Unexpectedly, *Tnf*, *Il6*, *Csf1*, *Ccl2* and *Cxcl1* were all elevated in the liver (Fig. 5c) of SPF vs. GF mice. These findings are in line with recent seminal reports demonstrating that commensal gut microbiota derived ligands translocate into the circulation under physiologic conditions to directly stimulate the liver innate-immune response in health^45, 46^. Corroborating the increased innate-immune cytokine expression findings in liver, but not ileum of SPF mice (Fig. 5b,c), the frequency of CD11b^+^LY6G^−^F4/80^+^LY6C^hi^ (inflammatory monocyte) cells^47, 48^ was enhanced in the draining liver (celiac, portal) lymph nodes (LLNs), but not the mesenteric lymph nodes (MLNs) of SPF mice (Fig. 5e). The upregulated innate-immune cytokine expression in liver and inflammatory monocyte cell frequency in draining LLNs, but no differences in ileum and MLNs of SPF mice, led to the postulation that the commensal gut microbiota has osteoimmunomodulatory actions mediated through a previously unidentified Gut-Liver-Bone Axis (Fig. 5f).

As a means to substantiate the authors’ novel Gut-Liver-Bone axis theory, the expression of pattern-recognition receptors (toll-like receptors, NOD-like receptors) and critical downstream signal transduction factors were evaluated in SPF vs. GF livers (Supplementary Fig. S3). Toll-like receptor (*Tlr)2*, which primarily recognizes extracellular microbial cell wall components, and *Tlr3*, which detects viral nucleic acids, were upregulated in the liver of SPF vs. GF mice (Supplementary Fig. S3a). Findings that critical regulators of TLR2 mediated signal transduction (*Myd88*, *Tirap(Mal)*, *Irak4*) were increased in SPF livers, while there was no difference in indispensable regulators of TLR3 mediated signal transduction (*Ticam1(Trif)*) (Supplementary Fig. S3b), indicate that the commensal gut microbiota’s pro-inflammatory actions in the liver are primarily mediated through TLR2 signaling. Notably, this investigation supports findings from prior reports demonstrating that normal gut microbiota derived ligands translocate into the circulation under physiologic conditions to directly stimulate the liver innate-immune response in health^45, 46^. While pattern-recognition receptor signaling in the liver has been extensively investigated under pathophysiological states, the current report delineates that resident gut microbes substantially upregulate TLR2 signaling in the liver in health.

Congruent with the enhanced *Tnf* expression in marrow and liver (Fig. 5a,c), serum TNF levels were significantly elevated in SPF mice (Fig. 5g). Serum CSF1 was similar in SPF vs. GF mice (Fig. 5h), which is consistent with knowledge that hepatic/splenic macrophages tightly regulate circulating CSF1 levels via receptor-mediated endocytosis^49^.

### T_H_17, CD4^+^IL17^+^ and CD4^+^IFNγ^+^ cells are increased in SPF marrow

**Figure 6:**
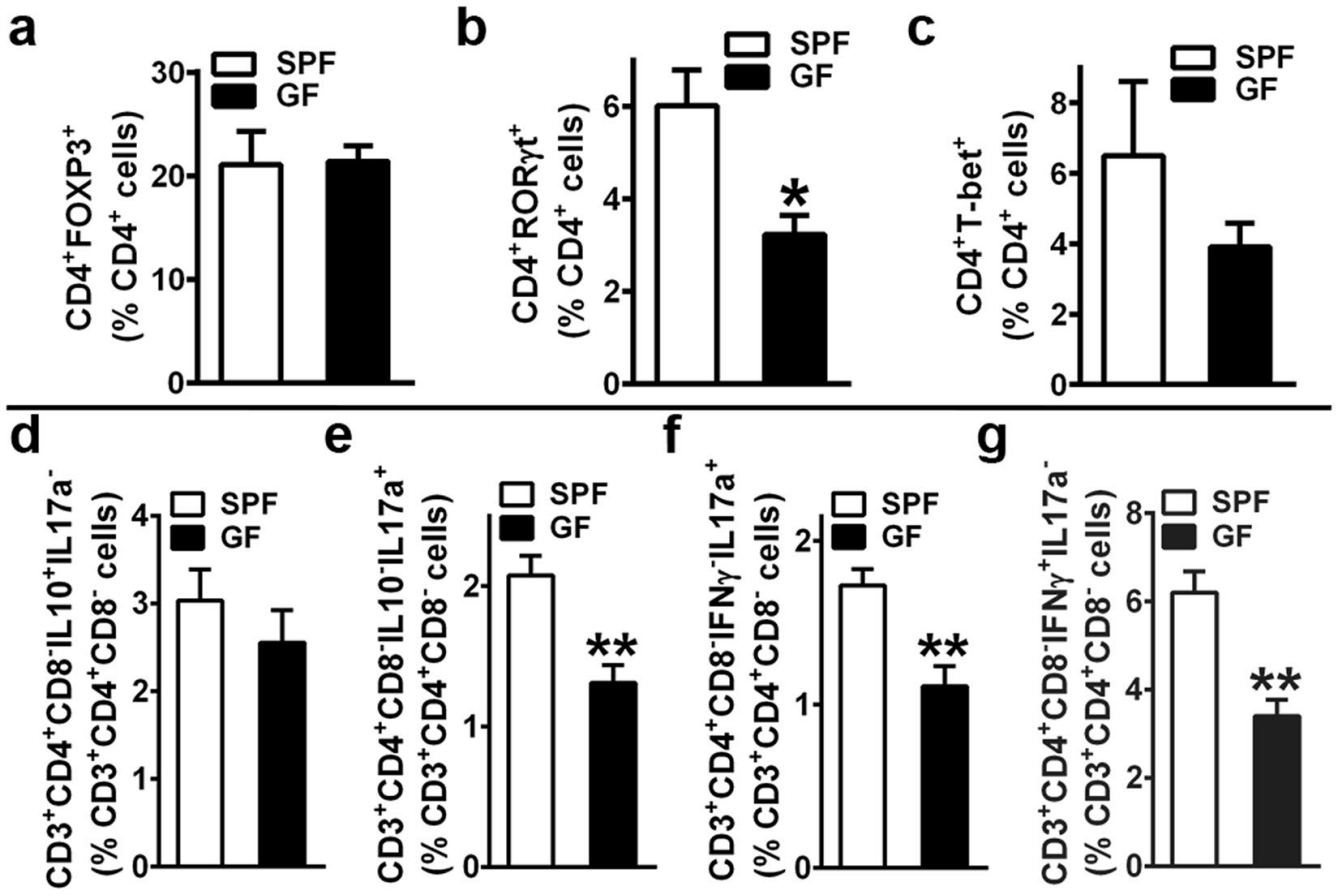
Bone marrow effector CD4^+^ T-cell hematopoiesis. (**a**–**c**) Marrow Transcription Factor Expression Assays: 12 week-old male SPF & GF mice were euthanized; femoral bone marrow cells were isolated and stained (n = 4/gp) for flow cytometric analysis to assess (**a**) % CD4^+^FOXP3^+^ (T_REG_) cells, (**b**) % CD4^+^RORγt^+^ (T_H_17) cells, and (**c**) % CD4^+^T-bet^+^ (T_H_1) cells. Percentages are expressed relative to CD4^+^ cells. (**d**–**g**) Marrow Intracellular Cytokine Expression Assays: 11 week-old gender matched (2 male and 2 female per group) SPF & GF mice were euthanized; femoral whole marrow was plated overnight for cytokine activation (PMA, Ionomycin, Monensin). Cells were isolated and stained (n = 4/gp) for flow cytometric analysis to assess (**d**) % CD3^+^CD4^+^CD8^−^IL10^+^IL17a^−^ (CD4^+^IL10^+^) cells, (**e**) % CD3^+^CD4^+^CD8^−^IL10^−^IL17a^+^ (CD4^+^IL17a^+^) cells, (**f**) % CD3^+^CD4^+^CD8^−^IFNγ^−^IL17a^+^ (CD4^+^IL17a^+^) cells, and (**g**) % CD3^+^CD4^+^CD8^−^IFNγ^+^IL17a^−^ (CD4^+^IFNγ^+^) cells. Percentages are expressed relative to CD3^+^CD4^+^CD8^−^ cells. Data are reported as mean ± SEM. *p < 0.05 vs. SPF; **p < 0.01 vs. SPF.

Despite knowledge that the commensal gut microbiota directs T-lymphocyte mediated immunity, the commensal gut microbiota immuno-regulatory effects on marrow CD4^+^/CD8^+^ T-cell hematopoiesis in the healthy adult skeleton is unclear. Recognizing the field of osteoimmunology has shown that specific bone marrow T-lymphocytic cells regulate osteoclastogenesis and bone remodeling in the adult skeleton^12–15^, flow cytometric analysis was employed to delineate alterations in these CD4^+^/CD8^+^ T-lymphocytic cell populations. While the overall frequency of marrow CD3^+^CD4^+^CD8^−^ (helper) T-cells and CD3^+^CD4^−^CD8^+^ (cytotoxic) T-cells were similar in SPF vs. GF mice (Supplementary Fig. S4), transcription factor expression analysis (Fig. 6a–c) and intracellular cytokine expression analysis (Fig. 6d–g) elucidated differences in the frequency of specific helper CD4^+^T-cell subsets and their characteristic cytokines.

Helper T-cell subsets of interest were based on the expression of characteristic cytokines known to regulate osteoclastogenesis: T_REG_ cells – IL10 (anti-osteoclastogenic); T_H_17 cells – IL17a (pro-osteoclastogenic); T_H_1 cells – IFNγ (anti-osteoclastogenic)^15^. Whereas there were no differences in the frequency of marrow CD4^+^FOXP3^+^ (T_REG_) cells (Fig. 6a) or CD4^+^T-bet^+^ (T_H_1) cells (Fig. 6c), the frequency of marrow CD4^+^RORγt^+^ (T_H_17) cells (Fig. 6b) was increased SPF vs. GF mice. Consistent with a greater frequency of T_H_17 cells (Fig. 6b), intracellular cytokine expression analysis revealed a higher frequency of CD3^+^CD4^+^CD8^−^IL10^−^IL17a^+^/CD3^+^CD4^+^CD8^−^IFNγ^−^IL17a^+^ (CD4^+^IL17a^+^) cells (Fig. 6e,f) in SPF marrow. Considering the lack of alterations in marrow T_H_1 cells in SPF vs. GF mice (Fig. 6c), the upregulated frequency of CD3^+^CD4^+^CD8^−^IFNγ^+^IL17a^−^ (CD4^+^IFNγ^+^) cells (Fig. 6g) in SPF marrow is likely attributed to IFNγ expressing non-T_H_1 helper T-cells. Intracellular cytokine expression analysis was also carried out in marrow cytotoxic CD8^+^T-cells to rule out alterations in T_C_17 and T_C_1 cell derived IL17a (CD8^+^IL17a^+^ cells) and IFNγ (CD8^+^IFNγ^+^ cells) (Supplementary Fig. S5).

While a recent experimental postmenopausal osteoporosis study in C57BL/6 SPF vs. GF mice demonstrated the commensal gut microbiota induces the upregulation of marrow TNF, IL17a, and IFNγ in sex steroid deprivation states^28^, the current study reveals the commensal gut microbiota enhances marrow T-lymphocyte expression of osteoclastogenic cytokines in health. The flow cytometry findings demonstrate the complex nature of lymphocytic cell interactions with bone cells in the marrow environment, providing novel insight into commensal microbiota *in vivo* immuno-stimulatory effects which could have altered the osteoclast maturation phenotype found in the *in vitro* OCP system (Fig. 4). Considering that IFNγ suppresses osteoclastogenesis through inhibition of RANKL-signaling via direct targeting of maturing osteoclast precursors^50, 51^, the suppressed sensitivity to RANKL-stimulation observed in day-5 SPF CD11b^neg^ OCP cultures (Fig. 4i-l) may be attributed to the enhanced frequency of marrow CD4^+^IFNγ^+^ cells in SPF vs. GF mice (Fig. 6g). Recognizing that IL17a is a pro-resorptive cytokine having potent synergistic effects on TNF pro-osteoclastic actions^14, 52^, the higher marrow *Tnf* (Fig. 5a)/circulating TNF (Fig. 5g) levels and increased frequency of marrow CD4^+^IL17a^+^ cells (Fig. 6e,f) in SPF vs. GF mice may mediate the enhanced terminal osteoclast maturation potential exhibited in day-7 SPF OCP cultures (Fig. 4m–p).

### Pro-inflammatory effector helper T-cells are increased in LLNs, but not MLNs of SPF mice

**Figure 7:**
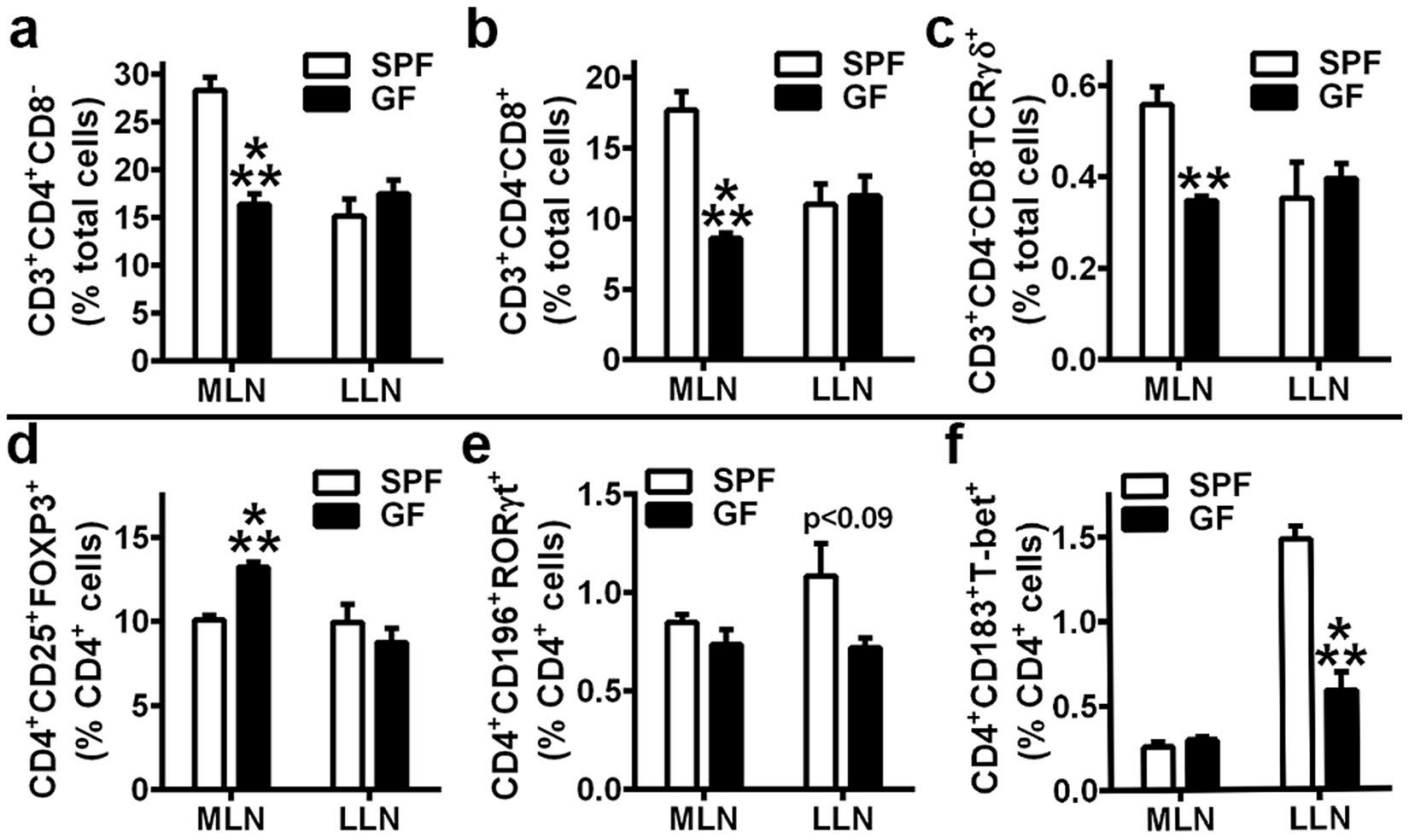
Draining gut and liver lymph node T-cell hematopoiesis. (**a**–**f**) 12 week-old male SPF & GF mice were euthanized; Mesenteric lymph node (MLN) and liver lymph node (LLN) cells were isolated and stained for flow cytometric analysis (n = 4/gp). (**a**) % CD3^+^CD4^+^CD8^−^ (helper) T-cells, (**b**) % CD3^+^CD4^−^CD8^+^ (cytotoxic) T-cells, and (**c**) % CD3^+^CD4^−^CD8^−^TCRγδ^+^ (gamma delta) T-cells are expressed relative to total lymph node cells. (**d**) % CD4^+^CD25^+^FOXP3^+^ (T_REG_) cells, (**e**) % CD4^+^CD196^+^RORγt^+^ (T_H_17) cells, and (**f**) % CD4^+^CD183^+^T-bet^+^ (T_H_1) cells are expressed relative to CD4^+^ cells. Data are reported as mean ± SEM. **p < 0.01 vs. SPF; ***p < 0.001 vs. SPF.

Microbial-associated molecular patterns (MAMPs) derived from normal gut microbiota structural components (lipopolysaccharide, peptidoglycan, nucleic acids, etc.) are recognized by host cells via pattern-recognition receptors (toll-like receptors, NOD-like receptors, etc.). Central to the authors’ postulated novel Gut-Bone-Liver Axis (Fig. 5f), normal gut microbiota derived (MAMP) byproducts circulating through liver sinusoidal vessels have been shown to induce innate-immune signaling at liver sinusoid endothelial cells and closely residing tissue resident Kupffer (liver tissue macrophage) and dendritic cells^45, 46^. Considering tissue resident dendritic cells in gut and liver present antigens in draining lymph nodes to regulate the commitment of naïve T-cells to effector T-cell subsets, T-cell hematopoiesis was assessed in LLNs and MLNs (Fig. 7) to further explore the commensal gut microbiota osteoimmunomodulatory effects mediated through hepato-gastrointestinal tissues.

Consistent with prior reports that lymphocytic cells are reduced in the gastrointestinal tissues of GF vs. SPF mice^1, 53, 54^, the frequency of overall CD3^+^CD4^+^CD8^−^ (helper) T-cells, CD3^+^CD4^−^CD8^+^ (cytotoxic) T-cells, and CD3^+^CD4^−^CD8^-^TCRγδ^+^ (gamma-delta) T-cells were decreased in the MLNs of GF mice (Fig. 7a–c). Transcription factor expression analysis of lymph node cells (Fig. 7d–f) was supplemented with cell surface activation markers, due to experimental design not including intracellular cytokine expression analysis. In line with a previous investigation in SPF vs GF mice^54^, the frequency of CD4^+^CD25^+^FOXP3^+^ (T_REG_) cells were upregulated in MLNs from GF mice (Fig. 7d), which may be attributed to the recognition of self-antigens or a hypersensitivity to food antigens in the absence of commensal gut microbiota immuno-stimulation. While there were no differences in the MLNs, the frequencies of CD4^+^CD196^+^RORγt^+^ (T_H_17) cells and CD4^+^183^+^T-bet^+^ (T_H_1) cells were marginally (Fig. 7e) and significantly (Fig. 7f) increased in the LLNs of SPF vs. GF mice.

The lack of alterations in T_H_1/T_H_17 cells in SPF MLNs is consistent with current knowledge the MLNs function as the primary site for oral tolerance induction to commensal gut microbiota antigens^55, 56^. In view of indirect evidence that supra-physiologic T_H_1/T_H_17 cell priming in MLNs drives pathophysiologic inflammation in gastrointestinal disease states^56, 57^, the finding that SPF mice have similarly increased effector helper T-cell populations in LLNs suggests that commensal gut microbiota pro-inflammatory actions in health are mediated through the liver. The realization that resident gut microbes induce sustained alterations in the adaptive-immune response in draining LLNs, but not MLNs of SPF mice, supports the authors’ postulate that the commensal gut microbiota has osteoimmunomodulatory actions mediated through a previously unidentified Gut-Liver-Bone Axis.

### GF mice colonization with SPF mice gut microbiota normalizes osteoimmunomodulatory outcomes

**Figure 8:**
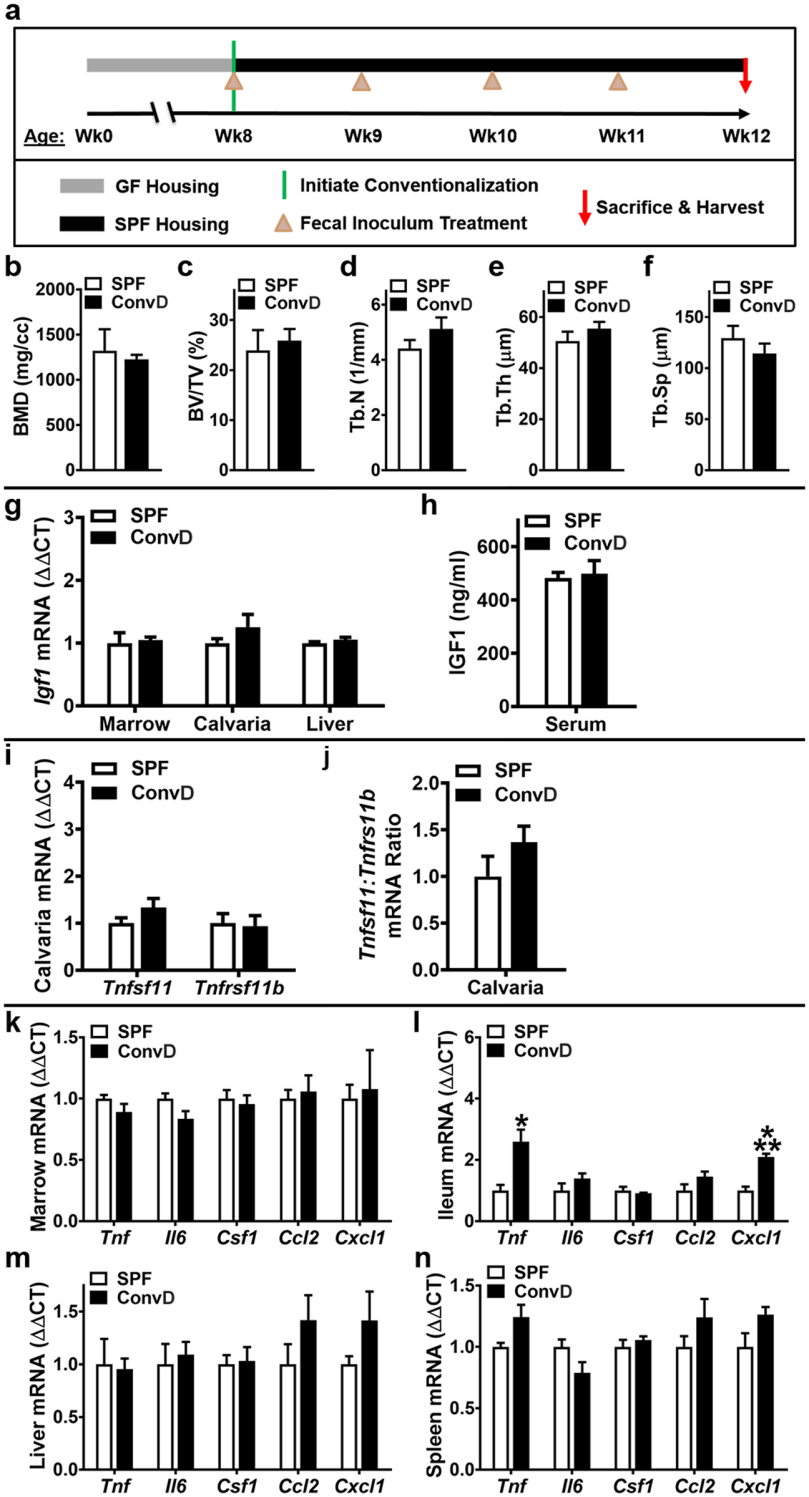
Conventionalization study: GF mice colonized with SPF mice gut microbiota. (**a)** Conventionalization study timeline: Conventionalized (ConvD) mice were generated by transferring 8 week-old male GF C57BL/6 littermate mice from sterile isolator housing to ventilated cages in a SPF vivarium. Microbial association was performed via fecal inoculum derived from pooled fresh feces of age matched male SPF C57BL/6 littermate mice, which were housed in the same SPF vivarium. (**b**–**n**) 12 week-old male SPF & ConvD mice were euthanized; tissues were harvested for analyses. (**b**–**f**) Trabecular bone analysis: Micro-CT analysis of proximal tibia trabecular bone (n = 4/gp). (**b**) BMD = trabecular bone mineral density. (**c**) BV/TV = trabecular bone volume fraction. (**d**) Tb.N = trabecular number. (**e**) Tb.Th = trabecular thickness. (**f**) Tb.Sp = trabecular separation. (**g**–**h**) *In vivo* regulation of osteoblastogenesis: (**g**) RNA was isolated from marrow (n = 4/gp), calvaria (n = 4/gp), liver (n = 4/gp) for qRT-PCR analysis. *Igf1* mRNA assessed as a critical osteoblastic signaling factor. Relative quantification of mRNA was performed via the comparative *C*_T_ method (ΔΔCT); *Gapdh* was utilized as an internal control gene; data expressed as fold difference relative to SPF. (**h**) Serum was isolated from whole blood (n = 4/gp); ELISA analysis of IGF1 levels. (**i**–**j**) *In vivo* regulation of osteoclastogenesis **:** qRT-PCR analysis in calvaria to assess alterations in the RANKL/OPG Axis. (**i**) *Tnfsf11(Rankl)* and *Tnfrsf11b(Opg)* mRNA levels. (**j**) *Tnfsf11(Rankl)*:*Tnfrsf11b(Opg)* ratio. Relative quantification of mRNA was performed via the comparative *C*_T_ method (ΔΔCT); *Gapdh* was utilized as an internal control gene; data expressed as fold difference relative to SPF. (**k**–**n**) *In vivo* pro-inflammatory cytokine expression: RNA was isolated from tissues and qRT-PCR analysis was performed in (**k**) bone marrow (n = 4/gp), (**l**) ileum (n = 4/gp), (**m**) liver (n = 4/gp), (**n**) spleen (n = 4/gp) to assess *Tnf*, *Il6*, *Csf1*, *Ccl2*, *Cxcl1* mRNA. Relative quantification of mRNA was performed via the comparative *C*_T_ method (ΔΔCT); *Gapdh* was utilized as an internal control gene; data expressed as fold difference relative to SPF. Data are reported as mean ± SEM. *p < 0.05 vs. SPF; ***p < 0.001 vs. SPF.

A conventionalization study was executed to validate that the commensal gut microbiota mediates the osteoimmunomodulatory effects found in the SPF vs. GF mouse model (Figs 1–7). Conventionalized (ConvD) mice were generated by microbially associating 8 week-old GF C57BL/6 littermate mice with gut microbiota from age matched SPF C57BL/6 littermate mice (Fig. 8a). Four weeks following conventionalization there were no differences in trabecular bone (Fig. 8b–f), critical osteoblastic signaling factors (Fig. 8g,h), or critical osteoclastic signaling factors (Fig. 8i,j) in ConvD vs SPF mice. Pro-inflammatory innate-immune cytokines normalized in the marrow (Fig. 8k) and liver (Fig. 8m) of ConvD mice. Findings that *Tnf* and *Cxcl1* were upregulated in the ileum of ConvD vs SPF mice (Fig. 8l) is in line with an acute innate immune response to the recently associated microbiota colonizing the proximal gut tissues, which is likely secondary to a transient increase in neutrophils expressing high levels of TNF and CXCL1.

## Discussion

This osteoimmunology study in young adult mice introduces the commensal gut microbiota as a critical immunoregulator of osteoclast-osteoblast mediated bone remodeling in the healthy adult skeleton. As opposed to prior investigations of the normal gut microbiota’s impact on physiological skeletal growth^19–23^ and pathophysiological skeletal deterioration^28–30^, this report is the first to delineate the commensal gut microbiota’s osteoimmunomodulatory effects on both osteoclastogenesis and osteoblastogenesis in the remodeling healthy adult skeleton.

Earlier investigations of the normal gut microbiota’s influence on skeletal physiology in growing GF mouse models are essentially different than the current investigation of the normal gut microbiota’s impact on bone remodeling in young adult GF mice. To properly appreciate our study outcomes, it is necessary to understand that bone modeling (growth) in the developing skeleton is a fundamentally different skeletal metabolic process than bone remodeling (turnover) in the adult skeleton. Sjogren *et al*.^21^ reported the normal gut microbiota has inhibitory effects on bone mass accrual in the growing skeleton of C57BL/6 Conv vs. GF mice, which the authors attributed to increased osteoclast numbers secondary to immunostimulatory effects in the gut. In contrast, more recent work showing that the normal gut microbiota supports skeletal growth in BALB/c Conv vs. GF mice^22^, highlights the potential influence of mouse strain genetic background on gut microbiota osteoimmunoregulatory effects. Investigations in BALB/c Conv vs. GF mice^22^ and CB6F1 (mixed BALB/c and C57BL/6 background) ConvD vs. GF mice^23^ delineated that the normal gut microbiota enhances skeletal growth and bone formation through pro-anabolic actions upregulating liver and serum IGF1. While the current report and prior osteoimmunology investigations in the GF murine model focus on commensal gut microbiota induced host immune response effects, it should be emphasized that confounding study variables (e.g., maternal care effects, environmental factors, diet, dissimilar microbiota across SPF housing facilities, etc.) potentially impact outcomes reported within/across studies.

The current investigation reveals that the normal gut microbiota has catabolic effects on skeletal homeostasis in the healthy adult skeleton, secondary to immunomodulatory actions suppressing osteoblastogenesis and enhancing osteoclastogenesis (Supplementary Fig. S6). SPF mice exhibited blunted bone formation *in vivo*, and reduced osteoblastic cell differentiation and mineralization *in vitro*, demonstrating the commensal microbiota has anti-anabolic effects on bone remodeling. Decreased *Sp7(Osterix)* in and *Igf1* in bone tissues and IGF1 in serum from SPF mice, indicate the commensal microbiota’s anti-osteoblastic actions are potentially mediated via suppression of local IGF1-signaling in skeletal tissues. Considering that prior investigations of mice on the BALB/c background have reported the gut microbiota enhances skeletal growth via upregulating liver derived IGF1^22, 23^, current study findings demonstrating that resident gut microbes suppress skeletal tissue derived IGF1 in C57BL/6 mice highlights IGF1 as a potential genetic determinant modulating the gut microbiota’s osteoimmunomodulatory effects. These findings are in line with prior investigations delineating that mouse strain background significantly impacts skeletal physiology^58, 59^. Knowledge that BALB/c and C57BL/6 mice differ in polyreactive IgA abundance which impacts the generation of antigen-specific IgA and microbiota diversity^60^, provides indirect evidence that genetic influence on host – microbiota interactions may have implications for the gut microbiota impact on osteoimmunology.

While Sjogren *et al*.^21^ reported the normal gut microbiota increases osteoclast numbers in growing Conv vs. GF mice, the current study has elucidated the commensal gut microbiota pro-catabolic effects on bone remodeling in the adult skeleton are secondary to immunomodulatory actions enhancing osteoclast size/maturation. Upregulated *Tnfsf11:Tnfrsf11b* (*Rankl:Opg*) ratio *in vivo*, and superior RANKL-stimulated terminal OCP maturation outcomes *in vitro*, in SPF vs. GF mice delineated that commensal gut microbiota pro-osteoclastic actions are related to sustained alterations in RANKL-signaling. Marrow *Tnf*, T_H_17 cells, and CD4^+^IL17a^+^ T-cells were increased in SPF mice, implying the commensal microbiota’s pro-osteoclastic actions are secondary to immunomodulatory effects directing marrow effector CD4^+^ T-cell hematopoiesis. Unexpected findings that SPF mice had upregulated pro-inflammatory innate-immune cytokines in liver, and increased pro-inflammatory innate- and adaptive-immune cells in LLNs, suggests the commensal gut microbiota’s osteoimmunomodulatory actions are partly mediated by a previously unidentified Gut-Liver-Bone Axis (Supplementary Fig. S6).

Appreciating that all of the toll-like receptors (TLRs) are expressed in the liver, and that TLR2, TLR4, TLR5 and TLR9 recognize the majority of gut microbiota derived ligands in the portal blood^45, 61^, future research is necessary to determine how signaling at specific TLRs in the liver regulates the normal gut microbiota’s osteoimmunomodulatory effects. Recognizing that distinct commensal gut bacteria disproportionately modulate the host immune response^1–5^, ongoing research is indicated to discern whether specific gut commensals drive the upregulated T_H_1/T_H_17 phenotype found in the LLNs and marrow of SPF mice. Considering indirect evidence that probiotic administration protects against estrogen depletion induced bone loss in experimental murine osteoporosis models^28, 62, 63^, non-invasive interventions (dietary modulation, pre/probiotics administration) in the gut microbiome need to be researched as a means to optimize skeletal tissue remodeling and homeostasis in health.

Findings from this investigation of the impact of the commensal gut microbiota vs. studies assessing the impact of pathophysiologic gastrointestinal conditions on skeletal metabolism underscore the necessity of understanding health when discerning mechanisms causing disease. Mechanisms that have been extensively reported to drive pathophysiologic bone loss in inflammatory hepato-gastrointestinal disease states, including increased liver CXCL1, CCL2, CSF1, IL6 and TNF, upregulated circulating TNF, decreased circulating IGF1, increased marrow T_H_17 cells/IL17a, unbalanced RANKL:OPG ratio, enhanced osteoclastogenesis, and blunted osteoblastogenesis^44, 64–68^, are astonishingly the same immunomodulatory mechanisms which appear to mediate the commensal gut microbiota’s catabolic effects on skeletal homeostasis in health.

This research defining the commensal gut microbiota immunomodulatory effects on osteoclast-osteoblast mediated skeletal remodeling in health calls into question what is “normal” bone remodeling, a phenomena that is currently poorly understood. Based on the generally accepted empirical theory governing bone remodeling, which dictates that osteoclastic-osteoblastic mediated actions are “coupled” (balanced) in health and “uncoupled” (unbalanced) in disease^12–14, 18^, findings reported here alarmingly imply the commensal gut microbiota induces a pathologic skeletal dys-homeostasis in health. The realization that the commensal gut microbiota has catabolic effects on endogenously programmed bone remodeling infers that bone remodeling is a not a simple “coupled” vs. “uncoupled” process, but rather a physiological gradient. The introduction of the commensal gut microbiota as a critical immunoregulator of normal osteoclast-osteoblast mediated bone remodeling processes in the healthy adult skeleton advances our understanding of skeletal physiology, having significant implications for the prevention of skeletal deterioration in health and disease.

## Methods

### Animals

Germ-free (GF) C57BL/6 mice were obtained from GF & Gnotobiotic Mouse Facilities at University of Michigan, and bred and maintained in sterile isolators at Medical University of South Carolina (MUSC) Gnotobiotic Animal Core. Specific-pathogen-free (SPF) C57BL/6 mice were purchased from Taconic, and maintained in ventilated cages in a SPF vivarium at MUSC. Mice were aged to 11–12 weeks for experiments. Animal procedures were approved by the MUSC Institutional Animal Care and Use Committee, and carried out in accordance with the approved guidelines.

### Conventionalization Study

Conventionalized (ConvD) mice were generated by transferring four 8 week-old GF C57BL/6 littermate mice from the MUSC Gnotobiotic Animal Core to the MUSC SPF vivarium facility. ConvD mice microbial association was performed via fecal inoculum derived from pooled fresh feces of four age matched SPF C57BL/6 littermate mice. Fresh feces from SPF mice was homogenized in PBS, and a fecal inoculum suspension was prepared at a concentration of 0.1 g/ml. At age 8 weeks, and weekly intervals thereafter until age 11 weeks, ConvD mice were administered fresh fecal inoculum (derived from age matched SPF mice) via oral gavage at an approximate dose of 200 uL per mouse; residual fecal inoculum suspension was deposited on the ConvD mice fur. Dirty bedding was transferred from SPF mice caging to ConvD mice caging during weekly inoculation treatments. ConvD and SPF mice were housed in the same SPF vivarium; cages were maintained side by side on the same rack, and on the same shelf during the entire procedure. Further precautions were taken to minimize cage effects, including that animal handling and cage changing/maintenance was carried out by designated personnel trained in gnotobiotic animal husbandry. One week following the final inoculation treatment, ConvD and SPF littermate mice were sacrificed at age 12 weeks.

### Histomorphometry

Femurs were fixed in 10% phosphate-buffered-formalin, dehydrated in graded EtOH and xylene, and embedded undecalcified in modified-methylmethacrylate^69^. Serial para-sagittal sections were cut through distal femur for trabecular bone analyses. 4 um tartrate-resistant acid phosphatase (TRAP) stained sections were used for quantifying osteoclast cellular endpoints and assessing eroded bone perimeter. 8 um sections were stained with toluidine blue for bone area analysis. 8 um unstained sections were used for dynamic indices of bone formation. 20 mg/kg calcein was administered via intraperitoneal injection 5 and 2 days prior to euthanasia^69^. Analyses were limited to the secondary spongiosa, beginning 250 µm proximal to the growth plate and extended 1000 µm proximally (50 µm from endocortical surfaces). Data were collected semi-automatically via an Olympus BX61 microscope and Visiopharm software. Data are reported in accordance with standardized nomenclature^70^.

### Micro-CT

Tibiae and femurs were fixed in 10% phosphate-buffered-formalin, and stored in 70% EtOH. Specimens were scanned with Scanco Medical µCT 40 Scanner, using the following acquisition parameters: X-ray tube potential = 55 kVp; X-ray intensity = 145 µA; Integration time = 200 ms; Isotropic voxel size = 6 µm^3^. Calibrated three-dimensional images were reconstructed. Tibia trabecular bone morphology was analyzed using Analyze 12.0 Bone Microarchitecture Analysis software (Analyze Direct). For trabecular analysis, transverse CT slices were analyzed beginning 250 µm distal to the proximal growth plate and extending 1200 µm distally; fixed threshold of 1750 Hounsfield Units was used to discriminate mineralized tissue. Femur length and cortical bone morphology were analyzed using Analyze 12.0 Bone Microarchitecture Analysis software (Analyze Direct). For cortical analysis, transverse CT slices were analyzed in a 1000 µm segment of the mid-diaphysis; fixed threshold of 2500 Hounsfield Units was used to discriminate mineralized tissue. Data are reported in accordance with standardized nomenclature^71^.

### Bone Marrow Cultures

Femurs and tibiae were harvested and marrow was flushed with α-MEM media, 10% FBS (Hyclone), 1% PSG (2 mM glutamine, 100 U/ml penicillin, 100 mg/ml streptomycin). For each animal, marrow cells were disassociated, counted, and plated at 3 × 10^6^ cells/cm^2^ in a 60 mm dish. 24-hours following plating, hematopoietic progenitor cells (HPCs) were isolated for osteoclast-precursor (OCP) assays via decanting off the non-adherent cells and culture media. Fresh media was added back to the bone marrow cultures, and 48-hours later adherent cells were resourcefully harvested for bone marrow stromal cell (BMSC) assays. Notably, marrow cells were not combined from animals for initial bone marrow cultures or subsequent OCP/BMSC assays; n-values reported for *in vitro* assays represent biological replicates.

### Osteoclast-Precursor (OCP) Assays

First passage HPCs (non-adherent cells isolated from bone marrow cultures) were washed, and incubated with CD11b MicroBeads (BD Biosciences). AutoMACS Sorter (Miltenyi Biotec) was applied to separate CD11b^neg^ HPCs, which were then stimulated in culture (primed with CSF1) to enrich for CD11b^neg^ osteoclast-precursor (OCP) cells having high osteoclastic potential^37–39^.

An initial sort of the HPCs was performed by CD11b positive selection using the Possel separation, which separated the HPCs into CD11b^hi^ and CD11b^low/neg^ populations. A subsequent sort of the CD11b^low/neg^ population was performed by CD11b positive selection using the Possel-s separation, which separated the CD11b^low/neg^ population into CD11b^low^ and CD11b^neg^ populations. CD11b^neg^ cells were washed, counted, and plated for assays in α-MEM media, 10% FBS (Hyclone), 1% PSG. *Gene expression assay:* CD11b^neg^ cells were plated at 1.0 × 10^5^ cells/cm^2^ in 12-well plates, and primed for 36 hours with 10 ng/ml CSF1 (R&D Systems) to drive the cells down the pre-osteoclastic lineage. CD11b^neg^ OCP cultures were then stimulated with fresh control (25 ng/ml CSF1; R&D Systems) or treatment (25 ng/ml CSF1 and 50 ng/ml RANKL; R&D Systems) media for 3 days; media was refreshed every other day. Day-3 control cultures (25 ng/ml CSF1) and treatment cultures (25 ng/ml CSF1 and 50 ng/ml RANKL) were harvested for quantitative real-time PCR (qRT-PCR) mRNA analysis. Gene expression assay was performed in duplicate (technical replicate) cultures. *TRAP stain assay:* CD11b^neg^ cells were plated at 1.5 × 10^5^ cells/cm^2^ in 96-well plates, and primed for 36 hours with 10 ng/ml CSF1 (R&D Systems) to drive the cells down the pre-osteoclastic lineage. CD11b^neg^ OCP cultures were then stimulated with fresh control (25 ng/ml CSF1; R&D Systems) or treatment (25 ng/ml CSF1 and 50 ng/ml RANKL; R&D Systems) media for 3, 5 and 7 days; media was refreshed every other day. Day-3/5/7 control cultures (25 ng/ml CSF1) and treatment cultures (25 ng/ml CSF1 and 50 ng/ml RANKL) were stained via the TRAP method, as previously reported^38, 39^. TRAP stain assays was performed in triplicate (technical replicate) cultures; three fields of view analyzed per technical replicate culture. TRAP + cell with > 3 nuclei considered an osteoclast.

### Bone Marrow Stromal Cell (BMSC) Assays

First passage BMSCs were isolated from bone marrow cultures; adherent cells were washed, trypsinized, counted, and plated for assays in α-MEM media, 10% FBS (Atlanta Biologicals), 1% PSG. Assays were performed in duplicate (technical replicate) cultures; medium changed every other day. *Cell expansion assay:* BMSCs plated at 2.0 × 10^4^ cells/cm^2^ in 24-well plates; untreated cultures harvested at day-1/3/5/7/9 for cell counts. *Differentiation potential assay:* BMSCs plated at 2.0 × 10^4^ cells/cm^2^ in 12-well plates; untreated pre-confluent (day-4) cultures were harvested for qRT-PCR mRNA analysis. Recognizing that cell confluency in the BMSC culture system drives the BMSCs down the osteoblastogenic lineage, cultures were harvested at a pre-confluent state to facilitate evaluating BMSC multipotent differentiation potential down the mesenchymal (osteoblastogenic, adipogenic, chrondrogenic) lineages. *Mineralization assay:* BMSCs plated at 1.0 × 10^5^ cells/cm^2^ in 12-well plates. Confluent (day-4) cultures were treated with mineralization medium (50 mg/ml ascorbic acid, 10 mM β-glycerophosphate) for 21 days. Mineralization detected by von Kossa method, as reported previously^69^.

### Quantitative Real-Time PCR (qRT-PCR) and NanoString

Whole calvaria, spleen, liver, and Ileum were flash frozen, pulverized, and homogenized in TRIzol Reagent (Invitrogen). Femur and tibia bone marrow were flushed with TRIzol. Cultures were washed twice with 1× PBS, and TRIzol was directly applied. RNA was isolated via the TRIzol method, following manufacturer’s protocol. Total RNA was quantified via NanoDrop 1000 (Thermo Scientific). *qRT-PCR gene expression analysis:* cDNA was synthesized from *in vitro* cell isolates and *in vivo* tissue isolates using Taqman Random Hexamers and Reverse Transcription Reagents, according to the manufacturer’s protocol. cDNA was amplified via the StepOnePlus System (Applied Biosystems) protocol, using TaqMan Universal PCR Master Mix and TaqMan gene expression primers/probes. Relative quantification of mRNA was performed via the comparative *C*_T_ method (ΔΔCT); *Gapdh* was utilized as an internal control gene^72^. *NanoString gene expression analysis:* nCounter PanCancer Immune Profiling for Mouse gene expression panel (NanoString Technologies) was applied to assess pattern-recognition receptor (PRR) signaling in liver. Hybridization was carried out and products were run on the nCounter preparation station, according to the manufacturer’s protocol. Data collected by the nCounter digital analyzer was evaluated via nSolver Analysis Software v2.6 (NanoString Technologies). Data were normalized to the geometric means of spiked-in positive controls and internal control genes. Absolute quantification of RNA reported as normalized RNA counts.

### Flow Cytometry

#### Live Cell Analysis

Mesenteric and liver (celiac, portal) lymph nodes cells were isolated, washed, and counted. Cells were treated with FcR-block (Miltenyi Biotec) and stains were performed. *Inflammatory monocytes:* anti-CD11b-APC (Miltenyi Biotec, clone M1/70.15.11.5), anti-Ly6g-PacB (Biolegend, clone 18A), anti-F4/80-PE (eBioscience, clone BM8), anti-Ly6c-FITC (Novus Biologicals, clone HK1.4). *T-cell hematopoiesis:* anti-CD3e-APC (Miltenyi Biotec, clone 145-2C11), anti-TCRγδ-e450 (eBioscience, clone eBioGL3), anti-CD8a-PE (Miltenyi Biotec, clone 53-6.7), anti-CD4-FITC (Miltenyi Biotec, clone GK1.5). Dead cells were excluded via propidium iodide viability dye (Miltenyi Biotec). Data was acquired by the MACSQuant System (Miltenyi Biotec). Analyses were performed via FlowJo VX software (TreeStar).

#### Transcription Factor Analysis

Femoral bone marrow, mesenteric and liver (celiac, portal) lymph nodes cells were isolated, washed, and counted. Cells were treated with FcR-block (Miltenyi Biotec), surface stains were performed and intracellular stains were carried out following the fixation-permeabilization buffer manufacturer’s protocol (eBioscience). *T*_REG_ *cells*: anti-CD4-FITC (Miltenyi Biotec, clone GK1.5), anti-CD25-PE (eBioscience, clone PC61.5), anti-FOXP3-APC (eBioscience, clone FJK-16s). *T*_*H*_ *17 cells:* anti-CD4-FITC (Miltenyi Biotec, clone GK1.5), anti-CD196-PE (Miltenyi Biotec, clone REA277), anti- RORγt-APC (Miltenyi Biotec, clone REA278). *T*_*H*_ *1 cells:* anti-CD4-FITC (Miltenyi Biotec, clone GK1.5), anti-CD183-PE (Miltenyi Biotec, clone CXCR3-173), anti-T-bet-APC (Miltenyi Biotec, clone REA102). Dead cells were excluded via e450 viability dye (eBioscience). Data was acquired by the MACSQuant System. ﻿Analyses were performed via FlowJo VX software.

#### Intracellular Cytokine Analysis

Femoral bone marrow cells were isolated and plated, consistent with methods reported for *In Vitro Assays*. Whole marrow cultures were maintained undisturbed for 4-hours; cultures were subsequently stimulated for 1-hour with 1 µl/ml PMA and 2 µl/ml Ionomycin, followed by 4-hours stimulation with 1 µl/ml Monensin. Cells were harvested, washed, and counted. Surface stains were performed and intracellular stains were carried out following the fixation-permeabilization buffer manufacturer’s protocol (BioLegend): anti-CD3e-FITC (Tonbo Biosciences, clone 145-2C11), anti-CD4-APC/Cy7 (BD Pharmingen, clone GK1.5), anti-CD8a-PerCP/Cy5.5 (BioLegend, clone 53-6.7), anti-IL10-APC (BioLegend, clone JES5-16E3), anti-IL17a-PE (eBioscience, clone eBio17B7), anti-IFNγ-BV421 (BioLegend, clone XMG1.2). Data was acquired by the FACSVerse System (BD Biosciences). Analyses were performed via FlowJo VX software.

### Serum Biochemical Assays

Whole blood was collected via cardiac puncture at euthanasia; serum was isolated, and stored at −80 °C. Mouse Quantikine ELISA Immunoassays were performed following manufacturer’s protocol (R&D Systems).

### Statistical Analysis

Unpaired *t* tests were performed using GraphPad Prism 6.0. Data are presented as mean ± SEM; significance is p < 0.05.

## Electronic supplementary material

Supplementary Information
