# Supplementary material for: Commensal Gut Microbiota Immunomodulatory Actions in Bone Marrow and Liver have Catabolic Effects on Skeletal Homeostasis in Health
Source: Sci Rep. 2017 Jul 18;7:5747. doi: 10.1038/s41598-017-06126-x (PMC5515851; doi:10.1038/s41598-017-06126-x)
Supplement: Supplementary file 1 — Supplementary Information [file 41598_2017_6126_MOESM1_ESM.pdf]

Commensal Gut Microbiota Immunomodulatory Actions in Bone Marrow and Liver have Catabolic Effects on Skeletal Homeostasis in Health

*Chad M. Novince[a], Carolyn R. Whittow[a], Johannes D. Aartun[a], Jessica D. Hathaway[a], Nicole Poulides[a], Michael B. Chavez[a], Heidi M. Steinkamp[a], Kaeleigh A. Kirkwood[a], Emily Huang[a], Caroline Westwater[a,b], Keith L. Kirkwood[a,b]

[a]Department of Oral Health Sciences and Center for Oral Health Research, College of Dental Medicine, Medical University of South Carolina, Charleston, South Carolina 29425; [b]Department of Microbiology and Immunology, Hollings Cancer Center, Medical University of South Carolina, Charleston, South Carolina 29425

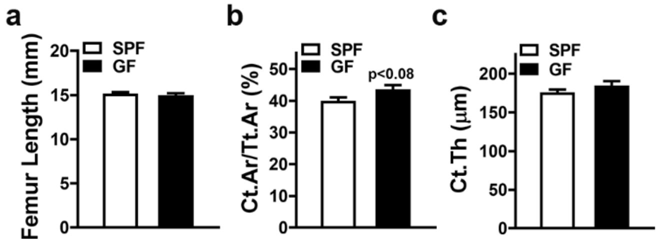

Supplementary Figure S1: Femur length and cortical bone analysis. (**a-c**) 11 to 12 week-old male SPF & GF mice were euthanized; femur harvested for micro-CT analysis (n=4/gp). (**a**) Micro-CT analysis of femur length. (**b,c**) Micro-CT analysis of femur mid-diaphysis cortical bone: (**b**) Ct.Ar/Tt.Ar = cortical area fraction; (**c**) Ct.Th = cortical thickness. Data are expressed as mean ± SEM.

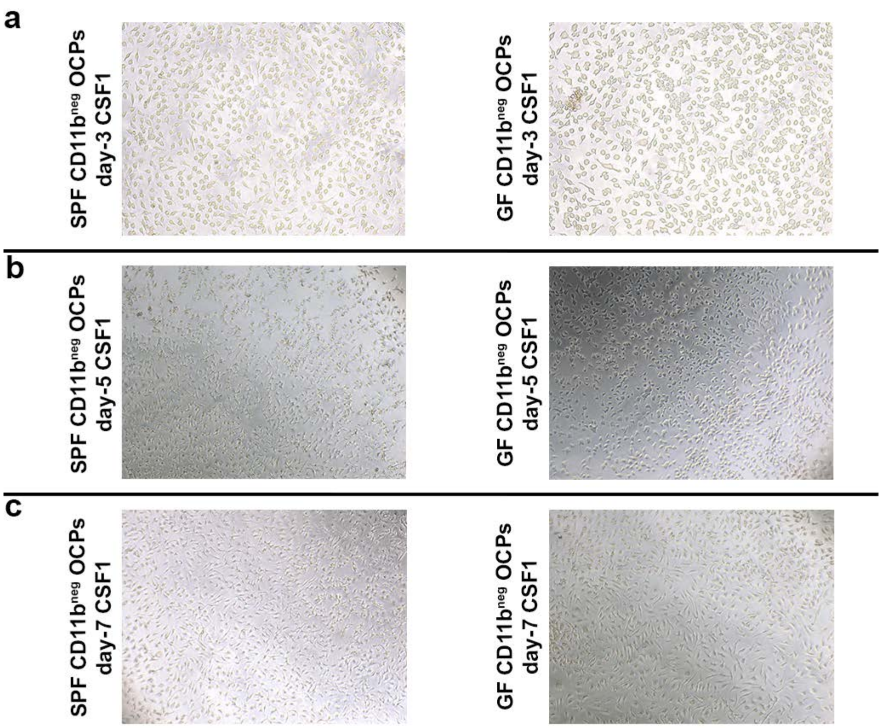

Supplementary Figure S2: Osteoclast-precursor (OCP) differentiation assays. (**a–c**) 11 week-old male SPF & GF mice were euthanized; bone marrow harvested; hematopoietic progenitor cells (HPCs) isolated. Magnetic cell sorting was applied to separate CD11b^{neg} HPCs, which were then stimulated in culture (primed with CSF1) to enrich for CD11b^{neg} osteoclast-precursor (OCP) cells having high osteoclastic potential. CD11b^{neg} OCP cultures were then stimulated with control (CSF1 alone) or treatment (CSF1 & RANKL) media for 3, 5 and 7 days. Cytomorphometric cellular differentiation endpoints were analyzed in TRAP stained CD11b^{neg} OCP cultures at day-3/5/7; TRAP+ cell with $\geq$3 nuclei considered an osteoclast. (**a**) Day-3 TRAP stain assay (n=4/gp). Representative images (200X) of CD11b^{neg} OCP cultures stimulated with control (CSF1) media for 3 days. (**b**) Day-5 TRAP stain assay (n=4/gp). Representative images (100X) of CD11b^{neg} OCP cultures stimulated with control (CSF1) media for 5 days. (**c**) Day-7 TRAP stain assay (n=4/gp). Representative images (100X) of CD11b^{neg} OCP cultures stimulated with control (CSF1) media for 7 days.

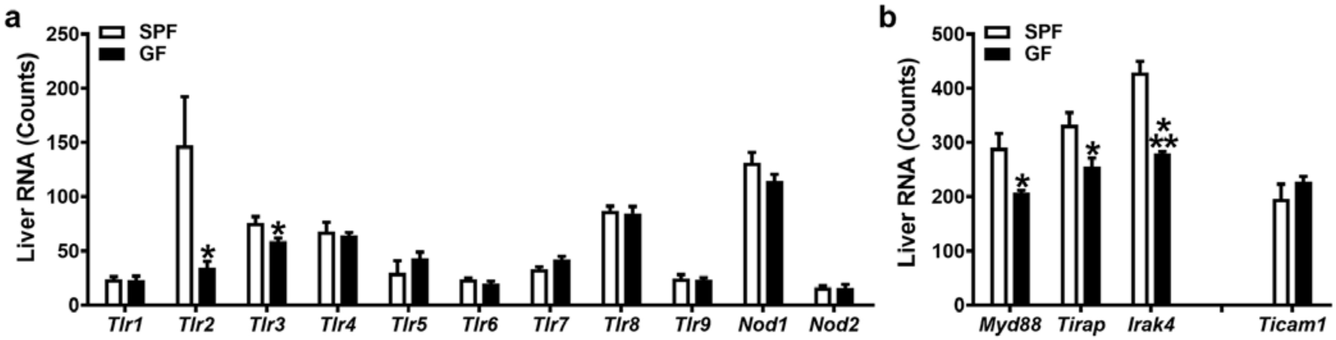

Supplementary Figure S3: Commensal microbiota *in vivo* regulation of pattern-recognition receptor (PRR) signaling in the liver. (**a,b**) 11 to 12 week-old male SPF & GF mice were euthanized; livers harvested for gene expression assays. RNA was isolated from livers (n=4/gp), and NanoString analysis was carried out to assess pattern-recognition receptor (PRR) signaling. (**a**) Analysis of toll-like receptor (TLR) and nucleotide-binding oligomerization domain (NOD) – like receptor levels: *Tlr1, Tlr2, Tlr3, Tlr4, Tlr5, Tlr6, Tlr7, Tlr8, Tlr9, Nod1, Nod2* RNA. (**b**) *Myd88, Tirap(Mal),* and *Irak4* RNA assessed as critical regulators of TLR2 mediated signal transduction; *Ticam1(Trif)* RNA assessed as a critical regulator of TLR3 mediated signal transduction. Data were normalized to the geometric means of spiked-in positive controls and internal control genes. Absolute quantification of RNA expressed as normalized RNA counts. Data reported as mean ± SEM. *p<0.05 vs. SPF; ***p<0.001 vs SPF.

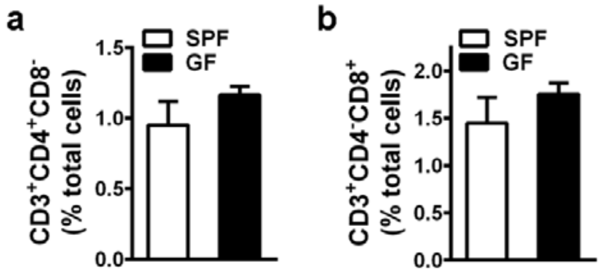

Supplementary Figure S4: Bone marrow T-cell hematopoiesis. (**a,b**) 11 week-old gender matched (2 male and 2 female per group) SPF & GF mice were euthanized; femoral whole bone marrow cells were isolated and stained for flow cytometry analysis (n=4/gp). (**a**) % $CD3^+CD4^+CD8^-$ (helper) T-cells. (**b**) % $CD3^+CD4^-CD8^+$ (cytotoxic) T-cells. Percentages are expressed relative to total marrow cells. Data are reported as mean ± SEM.

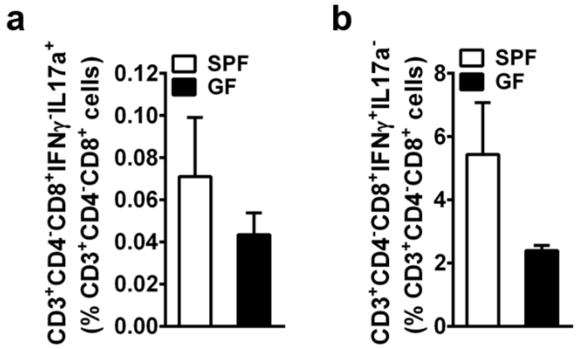

Supplementary Figure S5: Bone marrow effector $CD8^+$ T-cell intracellular cytokine analysis. (**a,b**) 11 week-old gender matched (2 male and 2 female per group) SPF & GF mice were euthanized; femoral whole bone marrow was plated overnight for cytokine activation (PMA, Ionomycin, Monensin); cells were isolated and stained for flow cytometric analysis of intracellular cytokine expression (n=4/gp). (**a**) % $CD3^+CD4^-CD8^+IFN\gamma^-IL17a^+$ ($CD8^+IL17a^+$) cells. (**b**) % $CD3^+CD4^-CD8^+IFN\gamma^+IL17a^-$ ($CD8^+IFN\gamma^+$) cells. Percentages are expressed relative to $CD3^+CD4^-CD8^+$ cells. Data reported as mean ± SEM.

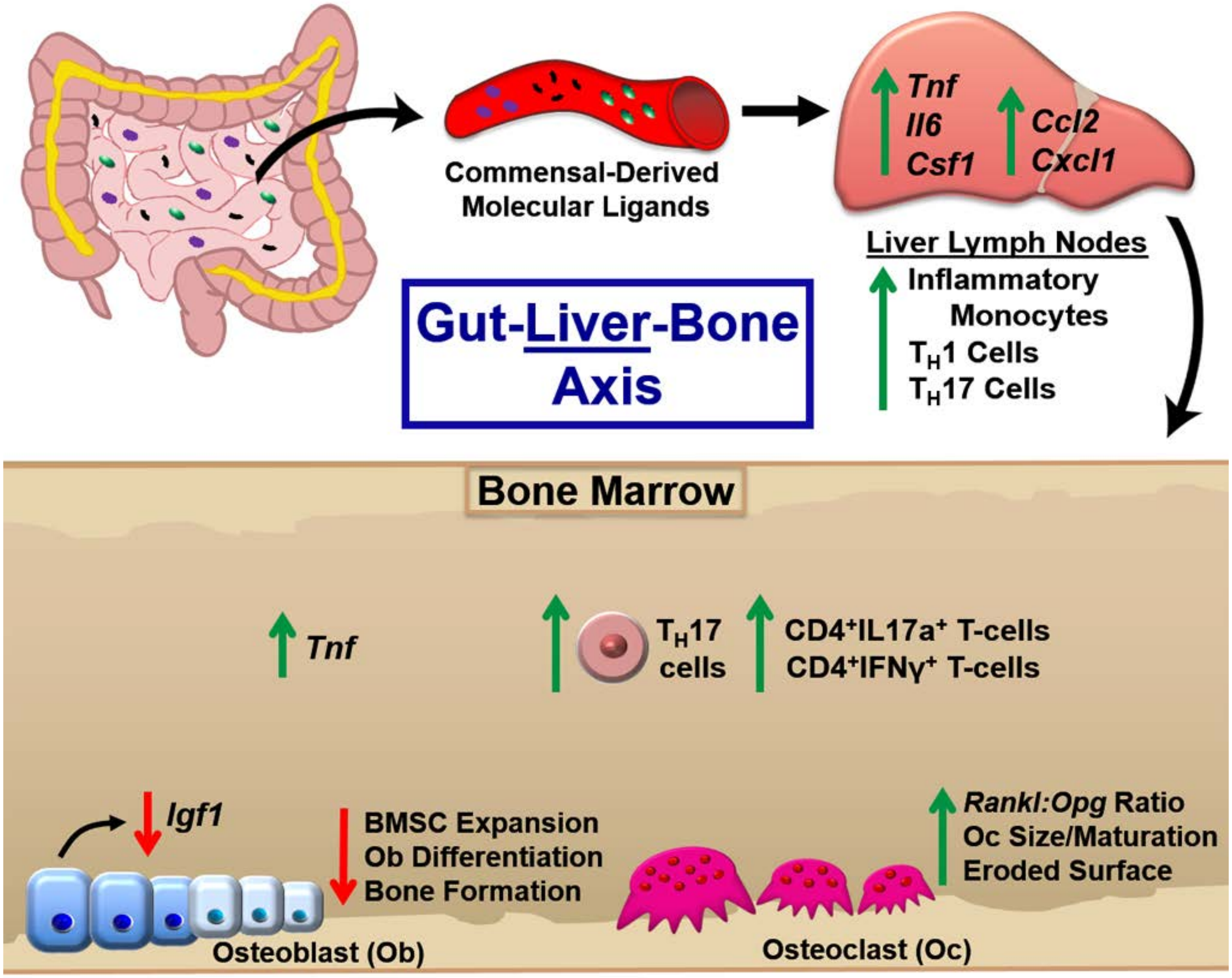

Supplementary Figure S6: Schematic summarizing study findings and proposed Gut-Liver-Bone Axis.
